# Extrinsic immune cell-derived, but not intrinsic oligodendroglial factors contribute to oligodendroglial differentiation block in multiple sclerosis

**DOI:** 10.1007/s00401-020-02217-8

**Published:** 2020-09-07

**Authors:** Laura Starost, Maren Lindner, Martin Herold, Yu Kang T. Xu, Hannes C. A. Drexler, Katharina Heß, Marc Ehrlich, Linda Ottoboni, Francesca Ruffini, Martin Stehling, Albrecht Röpke, Christian Thomas, Hans R. Schöler, Jack Antel, Jürgen Winkler, Gianvito Martino, Luisa Klotz, Tanja Kuhlmann

**Affiliations:** 1 Institute of Neuropathology, University Hospital Münster, Pottkamp 2, 48149 Münster, Germany; 2 Department of Cell and Developmental Biology, Max Planck Institute for Molecular Biomedicine, 48149 Münster, Germany; 3 Department of Neurology with Institute of Translational Neurology, University Hospital Münster, 48149 Münster, Germany; 4 The Solomon H. Snyder Department of Neuroscience, Johns Hopkins University School of Medicine, Baltimore, MD 21205 USA; 5 Bioanalytical Mass Spectrometry, Max Planck Institute for Molecular Biomedicine, 48149 Münster, Germany; 6 Neuroimmunology Unit, Institute of Experimental Neurology, Division of Neuroscience, IRCCS San Raffaele Hospital, 20132 Milan, Italy; 7 Institute of Human Genetics, University of Münster, 48149 Münster, Germany; 8 Montreal Neurologic Institute, McGill University, Montreal, QC H3A 2B4 Canada; 9 Department of Molecular Neurology, University Hospital Erlangen, Friedrich-Alexander-Universität Erlangen-Nürnberg, 91054 Erlangen, Germany; 10 Vita Salute San Raffaele University, 20132 Milan, Italy

**Keywords:** Induced pluripotent stem cells, Oligodendrocytes, Proteome, Relapsing–remitting multiple sclerosis, Remyelination, Ifnγ

## Abstract

Multiple sclerosis (MS) is the most frequent demyelinating disease in young adults and despite significant advances in immunotherapy, disease progression still cannot be prevented. Promotion of remyelination, an endogenous repair mechanism resulting in the formation of new myelin sheaths around demyelinated axons, represents a promising new treatment approach. However, remyelination frequently fails in MS lesions, which can in part be attributed to impaired differentiation of oligodendroglial progenitor cells into mature, myelinating oligodendrocytes. The reasons for impaired oligodendroglial differentiation and defective remyelination in MS are currently unknown. To determine whether intrinsic oligodendroglial factors contribute to impaired remyelination in relapsing–remitting MS (RRMS), we compared induced pluripotent stem cell-derived oligodendrocytes (hiOL) from RRMS patients and controls, among them two monozygous twin pairs discordant for MS. We found that hiOL from RRMS patients and controls were virtually indistinguishable with respect to remyelination-associated functions and proteomic composition. However, while analyzing the effect of extrinsic factors we discovered that supernatants of activated peripheral blood mononuclear cells (PBMCs) significantly inhibit oligodendroglial differentiation. In particular, we identified CD4^+^ T cells as mediators of impaired oligodendroglial differentiation; at least partly due to interferon-gamma secretion. Additionally, we observed that blocked oligodendroglial differentiation induced by PBMC supernatants could not be restored by application of oligodendroglial differentiation promoting drugs, whereas treatment of PBMCs with the immunomodulatory drug teriflunomide prior to supernatant collection partly rescued oligodendroglial differentiation. In summary, these data indicate that the oligodendroglial differentiation block is not due to intrinsic oligodendroglial factors but rather caused by the inflammatory environment in RRMS lesions which underlines the need for drug screening approaches taking the inflammatory environment into account. Combined, these findings may contribute to the development of new remyelination promoting strategies.

**Electronic supplementary material:**

The online version of this article (10.1007/s00401-020-02217-8) contains supplementary material, which is available to authorized users.

## Introduction

Multiple sclerosis (MS) is the most common inflammatory and demyelinating disease of the central nervous system (CNS) in Central Europe and North America. Symptoms depend on the localization and severity of MS lesions in the brain and spinal cord and, therefore, vary widely, but typically include numbness of limbs, vision problems, and fatigue [10]. The majority of patients (approximately 80%) start with a relapsing-remitting (RR) disease course characterized by episodes of acute exacerbation followed by complete or partial recovery [36]. Relapses are caused by newly formed inflammatory lesions which are characterized by loss of myelin, inflammation, gliosis, and axonal damage. The number of oligodendrocytes, the myelin-forming cells of the CNS, is relatively preserved in early lesion stages [37, 48] but decreases with lesion chronicity [40, 64]. Remyelination, the formation of new myelin sheaths around demyelinated axons, is an endogenous repair process which restores the conduction of action potentials, provides trophic support to the axon and protects against axonal injury and loss [24, 32, 52]. Remyelination requires the proliferation and migration of oligodendroglial precursor cells (OPCs) as well as their differentiation into mature oligodendrocytes [16]. In MS patients remyelination is associated with lower Expanded Disability Status Scale (EDSS) scores and slowed disease progression [6]; however, the extent of remyelination frequently decreases with disease duration and only approximately 20% of MS lesions are completely remyelinated [18, 46, 47]. Despite the frequent presence of OPCs at the borders of MS lesions, demyelinated axons often remain demyelinated due to an impaired migration and differentiation of OPCs into mature oligodendrocytes [8, 29, 63, 64]. MS-specific intrinsic oligodendroglial factors or an aberrant immune response may contribute to impaired remyelination. The outside-in model proposes that autoimmune-based inflammation is the primary event in MS leading to demyelination and ultimately to neurodegeneration, whereas the inside-out model suggests that oligodendroglial damage is the primary event causing inflammation and breakdown of myelin as it has been shown in animal models [20, 55, 57]. As access to primary human oligodendrocytes is very limited, further examination of these two possibilities is challenging.

Induced pluripotent stem cells (iPSCs) have emerged as a promising tool to overcome this obstacle, as they can give rise to all cell types of the human body including affected cells in a disease context [56]. During the last years, several protocols have been published which use iPSC-derived human oligodendrocytes to study demyelinating diseases [13, 14, 17, 61].

In the present study, we use a previously published protocol for the rapid and efficient generation of human iPSC-derived oligodendrocytes (hiOL) [14] to examine whether intrinsic oligodendroglial factors or the inflammatory milieu present in MS lesions contribute to impaired oligodendroglial differentiation. To this end, we generated and compared hiOL from three RRMS patients and three healthy controls, among them two monozygous twin pairs discordant for the disease, with respect to proliferation, migration, differentiation, in vitro myelination, and stress response. No significant differences were detected between RRMS hiOL and controls. Additionally, a global comparison of the proteome revealed highly similar proteomes of RRMS hiOL and controls. Moreover, to examine the effect of the inflammatory environment on oligodendrocytes in MS, we applied supernatants of activated peripheral blood mononuclear cells (PBMCs) to healthy and RRMS hiOL while differentiating. This resulted in a significant differentiation block, which was predominantly caused by interferon-gamma (IFNγ). Additionally, we found that drugs previously shown to promote oligodendroglial differentiation failed to restore impaired hiOL differentiation induced by PBMC supernatants, whereas immunomodulatory treatment of PBMCs partly restores hiOL differentiation indicating a demand for drug screening approaches mimicking the inflammatory environment in MS. In conclusion, our data suggest that the oligodendroglial differentiation block in RRMS is not mediated by intrinsic oligodendroglial factors but rather due to an inflammatory, extrinsic environment, which may contribute to impaired remyelination.

## Materials and methods

### Human tissue samples and cell lines

We retrospectively investigated paraffin-embedded brain tissue samples from 32 MS patients which were histologically characterized by inflammatory demyelination consisting with the histological diagnosis of MS. Patients underwent surgery after completion of extensive clinical diagnostic workup to exclude neoplastic or infectious diseases. The study was approved by the Ethics Committee of the University of Münster (AZ2012-407-f-S, 2016-165-f-S), with all methods performed in accordance with the relevant guidelines and regulations, and informed consent for surgery obtained from all patients. Information about MS patients is summarized in Supplementary Table 1, online resource. Patient numbers for individual histological analyses vary due to availability of brain tissue specimens.

Information about human cell lines is summarized in Supplementary Table 2, online resource. The study was approved by the local ethical committees in Münster, Erlangen and Milan (AZ 2018-040-f-S, AZ 259_17B and Banca INSpe).

### Generation of neural progenitor cells (NPCs)

Fibroblasts were isolated from skin biopsies from three RRMS patients and three healthy control individuals. Fibroblasts were reprogrammed into iPSCs by using the replication-incompetent Sendai virus kit (Invitrogen) according to the manufacturer’s instructions. Afterwards, NPCs have been generated as described earlier [49]. In brief, colonies of iPSCs were detached from mouse embryonic fibroblasts (MEFs) by treatment with 1 mg/mL collagenase IV. After sedimentation, cells were resuspended in human embryonic stem cell (hESC) medium without bFGF2 supplemented with 1 µM dorsomorphin (Tocris), 3 µM CHIR99021 (Axon Medchem), 10 µM SB-431542 (Ascent Scientific) and 0.5 µM purmorphamine (Alexis). Embryoid bodies (EBs) were formed by culturing cells in non-culture petri dishes (Corning). On day 2 medium was changed to N2B27 medium containing equal parts of neurobasal (Invitrogen) and DMEM-F12 medium (Invitrogen) with 1:100 B27 supplement lacking vitamin A (Invitrogen), 1:200 N2 supplement (Invitrogen), 1% penicillin/streptomycin/glutamine (PSG) and the same small molecules mentioned before. After two additional days dorsomorphin and SB-431542 were withdrawn, while ascorbic acid (AA; 150 µM) was added to the medium. On day 6, EBs were cut into smaller pieces and plated onto matrigel (Matrigel Growth-factor-reduced, Corning) coated 12-well plates. For passaging, NPCs were treated with accutase. After three passages purmorphamine was replaced by 0.5 µM SAG (Cayman Chemical) (NPC medium). NPCs were expanded until passage 10 before being infected for oligodendroglial differentiation. Three independent NPC generations were performed per iPSC line.

### Differentiation of human iPSC-derived oligodendrocytes

hiOL were differentiated as described previously [14, 62]. In short, NPCs were lentivirally transduced for 16 h with a polycistronic lentiviral vector containing the coding regions of human SOX10, OLIG2, NKX6.2 (SON factors) followed by an IRES-pac cassette allowing puromycin selection. After extensive washing and recovery in NPC medium, cells were cultivated in glial induction medium (GIM) consisting of DMEM-F12 with 1:100 B27 supplement lacking vitamin A, 1:200 N2 supplement, 1% PSG, 1 μM SAG, 10 ng/mL NT3 (Peprotech), 10 ng/mL PDGF-AA (Peprotech), 10 ng/mL IGF-1 (Peprotech), 200 μM AA (Sigma), 1:1000 Trace Elements B (Corning), 15 ng/mL T3 (Sigma) and 1 ng/mL bFGF-2 (Peprotech). The addition of GIM was termed as day 0 of differentiation. After 4 days differentiation medium (DM) comprising DMEM-F12 with 1:100 B27 supplement lacking vitamin A, 1:200 N2 supplement, 1% PSG, 10 ng/mL NT3, 50 µM dbcAMP (Sigma), 10 ng/mL IGF-1, 100 µM AA, 1:1000 Trace Elements B and 60 ng/mL T3 was added to the culture. Medium was changed every other day. At day 3 of differentiation, puromycin selection (0.75 µg/mL) was started for 5 days.

For differentiation of iPSC-derived oligodendrocytes based on an in vitro patterning protocol, 2.5 × 10^5^ NPCs were seeded onto a matrigel coated 12-well. The next day, pre-differentiation medium (PDM) comprising DMEM-F12 with 1:100 B27 supplement lacking vitamin A, 1:200 N2 supplement, 1% PSG, 1 μM SAG, 100 µM AA and 0.1 µM retinoic acid (RA) (Sigma) was added to the cultures, this day was termed as day 0 of differentiation. After 10 days, the medium was replaced by DM. Cells were differentiated until day 60 and replated at day 25 and 45 of differentiation.

### Differentiation of iPSC-derived neurons

For differentiation of iPSC-derived neurons, NPCs were seeded at densities of 1.5 × 10^5^ cells onto matrigel coated 12-well plates in NPC medium. The next day medium was replaced by N2B27 medium containing 1 µm SAG, 100 µM AA, 2 ng/mL BDNF (Peprotech) and 2 ng/mL GDNF (Peprotech). At day 6 medium was exchanged to N2B27 medium containing 100 µM AA, 2 ng/mL BDNF, 2 ng/mL GDNF, 1 ng/mL TGFβ (Peprotech) and 100 µM dbcAMP. From day 6 to 10 5 ng/mL Activin A were added to the medium. Cells were replated onto matrigel coated coverslips at day 10 of differentiation.

### Immunocytochemistry (ICC)

For ICC, cells were fixed in 4% paraformaldehyde (PFA). Afterwards, cells were permeabilized in 0.1% Triton for 10 min and blocked in 5% normal goat serum (NGS)/ 5% fetal calf serum (FCS) or 10% FCS (for SOX1 antibody) for 30 min. The permeabilization step was omitted when cells were stained for O4. Afterwards, cells were incubated with the primary antibody in the blocking solution overnight in the refrigerator. After three washing steps, Alexa Fluor-conjugated secondary antibody was applied for 1 h at room temperature. Subsequently, cells were washed three times with PBS. DAPI was added in the second washing step to counterstain the nuclei. Cells on plastic cell culture plates were analyzed on a Leica DMI6000 B inverted microscope, while cells on glass coverslips were mounted in Dako Fluorescent Mounting Medium (Dako) and visualized on a Zeiss LSM700 confocal microscope. Applied primary antibodies are listed in Supplementary Table 3, online resource.

### Immunohistochemistry (IHC)

For IHC, tissue specimens were fixed in 4% PFA and embedded in paraffin. Biopsy tissues were cut in 4-µm-thick sections and stainings were performed by using the Dako REAL™ Detection System (#K5001, Dako) and an automated immunostainer (AutostainerLink 48, Dako). In short, sections were deparaffinized and intrinsic peroxidase activity was blocked by incubation with 5% H_2_O_2_ in PBS for 5 min afterwards. Primary antibodies were applied as listed in Supplementary Table 3, online resource. IHC was completed by using biotinylated secondary antibodies followed by incubation with streptavidin/peroxidase complex and development with diaminobenzidine. For quantifications, white matter lesions in cerebellum and cerebrum were analyzed. Lesions were identified by loss of MBP staining and remyelination was identified by thin, irregular formed myelin sheaths. Determination of lesion activity was performed using CD68 stainings according to Kuhlmann et al. [30]. Active lesions are characterized by the presence of macrophages/microglia throughout the sampled lesion area [30]. Extent of remyelination was quantified using a semiquantitative score: 0 = complete absence of remyelination or presence only at the lesion border making up less than 10% of the whole lesion area, 1 = individual oligodendrocytes extending remyelinating processes, 2 = patchy remyelination, 3 = remyelination throughout the samples lesion area. For quantifications, at least 20 fields were analyzed using a morphometric grid.

### Quantitative RT-PCR (qRT-PCR)

Total RNA was isolated from cells using the RNeasy mini Kit (QIAGEN) and reverse transcribed into cDNA using the High Capacity cDNA reverse Transcription Kit (Applied Biosystems) according to the manufacturer’s instructions. qRT-PCR was performed on an Applied Biosystems StepOne Plus real-time cycler (Applied Biosystems) using the Power SYBR Green PCR master mix (Applied Biosystems). The conditions were 2 min at 50 °C, 10 min at 95 °C, 40 repeats of 15 s at 95 °C and 1 min at 60 °C, followed by 15 s at 95 °C, 1 min at 60 °C and 15 s at 95 °C. To ensure specificity of each product melting curves were measured and product size was confirmed by agarose gel electrophoresis. All samples were performed as triplicates. Expression levels were calculated by using the 2-^ΔΔCt^ method and normalization was performed by using *GAPDH* as a reference gene. Applied primers are listed in Supplementary Table 4, online resource.

### Three germ layer differentiation

Three germ layer differentiation was performed as described previously [54]. Briefly, EBs were generated by cutting and detaching colonies of iPSCs seeded on MEFs. Afterwards, EBs were cultivated in non-culture petri dishes containing hESC medium supplemented with 1 µM dorsomorphin and 10 µM SB-431542. After 2 and 4 days medium was changed to hESC medium without additional supplements. After 6 days EBs were plated either onto matrigel coated 12-well plates in N2B27 medium for ectodermal differentiation or onto gelatine-coated plates in DMEM with 1% PSG and 20% FCS for mesodermal and endodermal differentiation. Medium was changed every 3 days and cells were fixed and stained for tissue-specific markers after 14 days.

### Karyotype analysis

For karyotype analysis, 0.1 mL colcemid solution (10 µg/mL KaryoMAX Colcemid solution; Gibco) was applied to iPSCs for 3 h. After incubation at 37 °C, cells were singularized by treatment with Trypsin–EDTA (0.05%; Gibco) and centrifuged. Subsequently, cell pellets were resuspended in prewarmed 75 mM KC solution and incubated for 7 min at 37 °C. Afterwards, cells were again centrifuged and resuspended in fresh fixation solution consisting of 3:1 methanol/acetic acid while shaking. After another centrifugation step, fixation solution was renewed and cells were incubated for 20 min at 4 °C followed by transfer of drops on glass slides (Menzel Gläser, Thermo Scientific) for analysis. Chromosomes were GTG-banded using standard procedures and metaphase spreads were analyzed using the slide scanning software Metafer (Metasystems, Altlussheim Germany).

### Flow cytometry

For flow cytometric analysis of oligodendroglial differentiation and cell death, hiOL were stained and quantified by using anti-O4-APC or Annexin V according to the manufacturer’s instruction (Miltenyi). Briefly, cells were singularized by treatment with accutase. After washing and separation with a 40 µm cell strainer, cell numbers were determined and anti-O4-APC or Annexin V were applied to the cells, which were then incubated either for 10 min in the dark in the refrigerator (O4) or for 15 min at RT (Annexin V). Cells were washed and subsequently analyzed onto a FACSAria IIIu cell sorter (BD Biosciences). O4^+^ hiOL that were identified by using unstained cells and isotype controls were immediately seeded in DM. Propidium iodide (PI) was added to mark dead cells for cell death assays. Gating strategy for O4^+^ cells was described previously [62]. For flow cytometric analysis of immune markers on differentiating hiOL, cells were singularized as described and incubated with antibodies against immune markers for 15 min at RT. Subsequently, cells were washed and fixed in 0.4% PFA for 20 min. Afterwards, fixed cells were analyzed on a FACSAria Fusion cell sorter (BD Biosciences). Multicolor compensation and gating were performed by utilizing single marker stainings and fluorescence minus one controls. Analysis was performed with FlowJo software (BD Biosciences). For flow cytometric analysis of PBMCs and related subgroups, surface marker staining was performed as described previously [28]. Analysis was performed using a Gallios Flow Cytometer (Beckman Coulter) and results were analyzed with Kaluza software (Beckman Coulter). Information on applied antibodies is summarized in Supplementary Table 3, online resource.

### Migration assay

Migrating cells were analyzed by using live-cell analyzer JuLI™ Br as well as xCELLigence Real-Time cell Analysis instrument. Therefore, O4^+^ RRMS and control hiOL were purified by flow cytometry at day 12 of differentiation and seeded onto Laminin LN521 (Biolamina) coated 12-well plates (for JuLI™ Br) or CIM-Plate 16 (for xCELLigence). For JuLI™ Br recording, cells were allowed to sit for 4 h followed by recordings of undirected moving for 24 h. Cells were tracked and analyzed using MTRACKJ plugin. For xCELLigence analysis, O4^+^ hiOL were seeded and impedance-based measurement was performed immediately for 16 h.

### Nanofiber assays

For in vitro myelination assay, O4^+^ RRMS and control hiOL were purified by flow cytometry at day 21 of differentiation. Afterwards, 1 × 10^5^ O4^+^ hiOL were seeded in DM onto aligned nanofibers (Nanofiber solutions) that were coated with 1 µg/mL PLO and 5 µg/mL Laminin LN211 (Biolamina) beforehand. For staining of nanofibers, coating was performed by replacing PLO with 100 µg/mL PLL-FITC (Sigma). Half of the medium was changed every other day. After 7 days, cells were fixed and stained for MBP. A previously published heuristic algorithm was used for quantification [66].

### Stress assays

O4^+^ RRMS and control hiOL were sorted at day 21 of differentiation and reseeded at densities of 7.5 × 10^3^ cells onto matrigel coated 96-well plates in DM. After 4 days of recovery, cells were stressed with either vehicle [0.01% (vol/vol) DMSO] or rotenone (100, 250, 500 nM), peroxynitrite (0.01, 0.05, 0.1 mM) (Merck), IFNγ (100, 200, 400 ng/mL) (Peprotech) or TNFα (50, 100, 200 ng/mL) (Peprotech). After 24 h (rotenone, peroxynitrite) or 48 h (IFNy, TNFα) cell viability was assessed by using CellTiter Glo (Promega) according to the manufacturer’s instruction.

### Liquid chromatography–mass spectrometry (LC–MS/MS) analysis

For LC–MS/MS analysis which was performed as described previously [39], O4^+^ RRMS and control hiOL or iPSC-derived oligodendrocytes were sorted at day 28 (hiOL) or day 60 (iPSC-derived oligodendrocytes differentiated by in vitro patterning approach) of differentiation and prepared for bottom-up MS analysis using a one-pot sample preparation method (iST Sample Preparation Kit; Preomics) following the manufacturer´s instructions with the exception of two additional fractionation steps prior to final elution of peptides. Briefly, subsequent to the final washing steps, microcolumns were first eluted with 100 µl SDB-RPSx1 (100 mM ammonium formate, 40% (v/v) acetonitrile, 0.5% (v/v) formic acid), then with 100 µl SDB-RPSx2 (150 mM Ammonium formate, 60% (v/v) Acetonitrile, 0.5% (v/v) Formic acid), and finally with 100 µl of the elution buffer provided with the kit [31]. hiOL samples were analyzed twice on an Easy nLC 1200 system coupled to a Q Exactive HF mass spectrometer by a Nanospray Flex ion source (ThermoFisher Scientific). Peptides were dissolved in buffer A (0.1% formic acid) and separated on a 25 cm column, in-house packed with 1.9 µm C18 beads (Reprosil -Pur C18 AQ, Dr. Maisch) using a multi-linear gradient from 2 to 20% buffer B (80% acetonitrile; 0.1% formic acid) within 150 min and from 20 to 50% B in 90 min followed by an increase to 90% B in 10 min, a final washout for 5 min again at 90% B and re-equilibration at starting conditions (100% buffer A; flow rate 250 nL/min). The Q-Exactive HF mass spectrometer was operated in data-dependent acquisition mode (spray voltage 2.1 kV; column temperature maintained at 45 °C using a PRSO-V1 column oven (Sonation)). MS1 scan resolution was set to 60.000 at m/z 200 and the mass range to m/z 300–1750. AGC target value was 3E6 with a maximum fill time of 100 ms. Fragmentation of peptides was achieved by Higher-energy collisional dissociation (HCD) using a top17 method (MS2 scan resolution 15.000 at 200 m/z; AGC Target value 1E5; maximum fill time 50 ms; isolation width 1.6 m/z; normalized collision energy 27). Dynamic exclusion of previously identified peptides was allowed and set to 20 s, singly charged and peptides assigned with charge of eight and more were excluded from the analysis. Data were recorded with Xcalibur software (Thermo Scientific). For the measurement of iPSC-derived oligodendrocytes that were generated by the in vitro patterning protocol, a Bruker nanoElute UHPLC system was coupled via a Captive Spray ion source to a hybrid TIMS-quadrupole time-of-flight mass spectrometer (tims-ToF Pro, Bruker Daltonics, Bremen, Germany). Peptide mixtures were dissolved in 0.1% formic acid (FA) and separated within 120 min with a linear gradient from 3 to 35% B on a self-packed C18 reverse-phase capillary column with pulled emitter tip (nanoseparations; 360 µm OD × 75 µm ID × 250 mm; Reprosil pur C18-aq, 1.9 µm, Dr. Maisch) using a constant flow of 300 nl/min (Buffer A: 0.1% formic acid; Buffer B: 0.1% FA, 80% ACN). At the end of the gradient the column was flushed with 90% B before re-equilibration at starting conditions. MS and MS/MS spectra were recorded in positive mode from m/z 100 to 1700 Da, using the PASEF scan mode. Each duty cycle consisted of 1 TIMS-MS and an average of 10 PASEF MS/MS frames, each one containing multiple MS/MS spectra, which resulted in a total cycle time of 1.1 s. To exclude the majority of singly charged ions with low m/z for PASEF precursor selection, a polygon filtering was applied to the m/z over ion mobility area. For the 120 min runs target intensity was set to of 20.000 cts/s and an ion mobility range (1/K0) of 0.6–1.6 Vs/cm^2^ was used. Data were acquired with a 100 ms ramp time. The Bruker Hystar / OTOF Control software was used to control the whole LC–MS system and record the data (version 3.2, Bruker Daltonics, Bremen; Germany).

### MS data analysis and label-free quantification

Raw MS files were processed using the MaxQuant computational platform (version 1.6.6.0) [11]. Identification of peptides and proteins was enabled by the built-in Andromeda search engine querying the concatenated forward and reverse mouse Uniprot database (UP000005640_9606.fasta; version from 04/2019) including common lab contaminants. Allowed initial mass deviation was set to 7 ppm and 20 ppm, respectively, in the search for precursor and fragment ions. Trypsin with full enzyme specificity and only peptides with a minimum length of seven amino acids were selected. A maximum of two missed cleavages was allowed; the ‘match between runs’ option was turned on. Carbamidomethylation (Cys) was set as a fixed modification, while Oxidation (Met) and *N*-acetylation at the protein N-terminus were defined as variable modifications. For peptide and protein identifications a minimum false discovery rate (FDR) of 1% was required.

Relative label-free quantification (LFQ) was based on the measurements of 2 × 3 biological replicates for RRMS and control hiOL. Data processing was performed using Perseus (version 1.6.6.0) [58]. First, reverse and contaminant hits, as well as proteins that were identified by a single modified peptide only, were eliminated from the list of identified protein groups. Proteins eventually included for further analysis had to be identified with at least two peptides, one of which had to be unique for the protein group. LFQ intensity values were log2 transformed and missing values (NaN) were replaced by imputation (downshift 1.8, width 0.3), simulating signals of low abundant proteins within the distribution of measured values. To identify in a supervised manner the sets of proteins that significantly distinguish control and RRMS hiOL, a two-sample *t* test with a permutation-based FDR of 0.05 to correct for multiple hypothesis testing was performed on the averaged log2 transformed LFQ values from both experiments. The overlap of proteins identified from both experimental groups was shown by a Venn diagram generated using the BioVenn web application [22].

Protein names and averaged log2 transformed LFQ values across all samples (control and RRMS hiOL) were loaded into the R environment (version 3.6.0). For further analysis, protein names were converted into Entrez IDs using the R package AnnotationDbi (version 1.46.1). For enrichment analysis, a curated list of cell markers was imported [68]. Enrichment analysis for Entrez IDs of proteins with log2 transformed LFQ > 35 (*n* = 167) was then performed using the *enrichr* function of the R package clusterProfiler (version 3.12.0) with a Benjamini–Hochberg correction and an adjusted *p* value of 0.05 [67]. Cell markers for recently described oligodendroglial subtypes OPC, COP, ImOLG, Oligo1, Oligo2, Oligo3, Oligo4, Oligo5 and Oligo6 derived from single-nucleus RNA sequencing of post-mortem human brain were imported from Jäkel et al. [23] (Supplementary Table 3: a list of differentially expressed genes for each of the 23 nuclei clusters). Relative intersections of all gene names from each cluster with all candidates from the hiOL experiments and candidates with mean log2 transformed LFQ > 35 were visualized using the geom_polar function of ggplot2 (version 3.2.1).

### Isolation of peripheral blood mononuclear cells (PBMCs)

Healthy donor PBMCs were obtained from the peripheral blood collected in leukoreduction system (LRS) chambers and isolated by Ficoll density gradient. Immune cell subsets were isolated using specialized isolation kits from Miltenyi Biotec. Purity was determined by flow cytometry. Freshly-isolated PBMCs were stimulated with 5 µg/ml phytohaemagglutinin (PHA) or left unstimulated for up to 48 h and cultivated in X-Vivo 15 medium (Lonza). Afterwards, supernatants were collected, aliquoted, and frozen at − 80 °C.

### Application of recombinant proteins, oligodendroglial differentiation promoting drugs, and PBMC supernatants to hiOL

Supernatants were applied from day 4 until day 21 of differentiation to hiOL for assessing the effects on early differentiation or from day 21 until day 35 of differentiation to untreated O4^+^ hiOL sorted at day 21 of differentiation for terminal differentiation. For iPSC-derived neurons, supernatants were applied from day 6 until day 23 of differentiation. Medium was changed every other day and supernatants were diluted 1:10 in DM or neuronal medium unless stated otherwise. For neutralization experiments, activated PBMC supernatants were incubated for 1 h at 37 °C with 50 µg/mL IgG control, 50 µg/mL anti-IFNγ, 50 µg/mL anti-TNFα antibody or combination of both while shaking. Subsequently, supernatants were diluted 1:10 in DM and applied to hiOL from day 4 of differentiation until day 21 for assessing early differentiation. Information about neutralization antibodies is summarized in Supplementary Table 3, online resource. Concentrations of TNFα and IFNy in the supernatants before and after neutralization were determined by ELISA according to manufacturer’s instructions (eBioscience). For heat-inactivation experiments, activated PBMC supernatants were incubated for 20 min at 60 °C prior to application to differentiating hiOL. Recombinant proteins of IFNγ (400 ng/mL), TNFα (200 ng/mL), or combination of both were applied to differentiating hiOL from day 4 until day 21 of differentiation. For testing of oligodendroglial differentiation promoting drugs, clemastine (1 µM, Selleckchem), benztropine (1 µM, Sigma), and miconazole (1 µM, Sigma) were added to differentiating hiOL that were simultaneously treated with activated PBMC supernatants in a dilution of 1:50 from day 4 until day 21 of differentiation or to untreated O4^+^ hiOL sorted at day 21 of differentiation that were simultaneously treated with activated PBMC supernatants in a dilution of 1:10 from day 21 until day 35 of differentiation. To investigate whether immunomodulatory treatment of PBMCs affects hiOL differentiation, PBMCs were treated with 100 µM teriflunomide (kindly provided by Sanofi Genzyme) with and without parallel PHA stimulation. After 48 h supernatants were collected and applied to hiOL from day 4 until day 21 of differentiation.

### Statistical analysis

All experiments comparing RRMS and control hiOL except proteomic analysis were performed at least once per NPC clone, thus at least three times for one iPSC line. For statistical analysis, the mean value from all NPC clones per single iPSC line was first determined and then pooled with the mean values of the other two lines of the same group (either control or RRMS). All statistics except for LC–MS/MS data analysis were calculated using the GraphPad Prism 5 or 8 software (GraphPad Software). To compare two groups, a Student's two-tailed *t* test was applied. For comparison of three or more groups, Bonferroni-corrected one-way or two-way ANOVA was performed. Data are presented as means + SEM (*p < 0.05, **p < 0.01, ***p < 0.001). Quantifications were either performed in a blinded or machine-learning-based manner.

### Data and code availability statements

All mass spectrometry proteomics data generated in this study were deposited to the ProteomeXchange Consortium (https://proteomecentral.proteomexchange.org) [60] via the PRIDE partner repository with the data set identifiers PXD016043 (hiOL) and PXD016043 (iPSC-derived oligodendrocytes generated by in vitro patterning approach).

## Results

### RRMS and control hiOL show the same proliferation and migration capacities

**Fig. 1:**
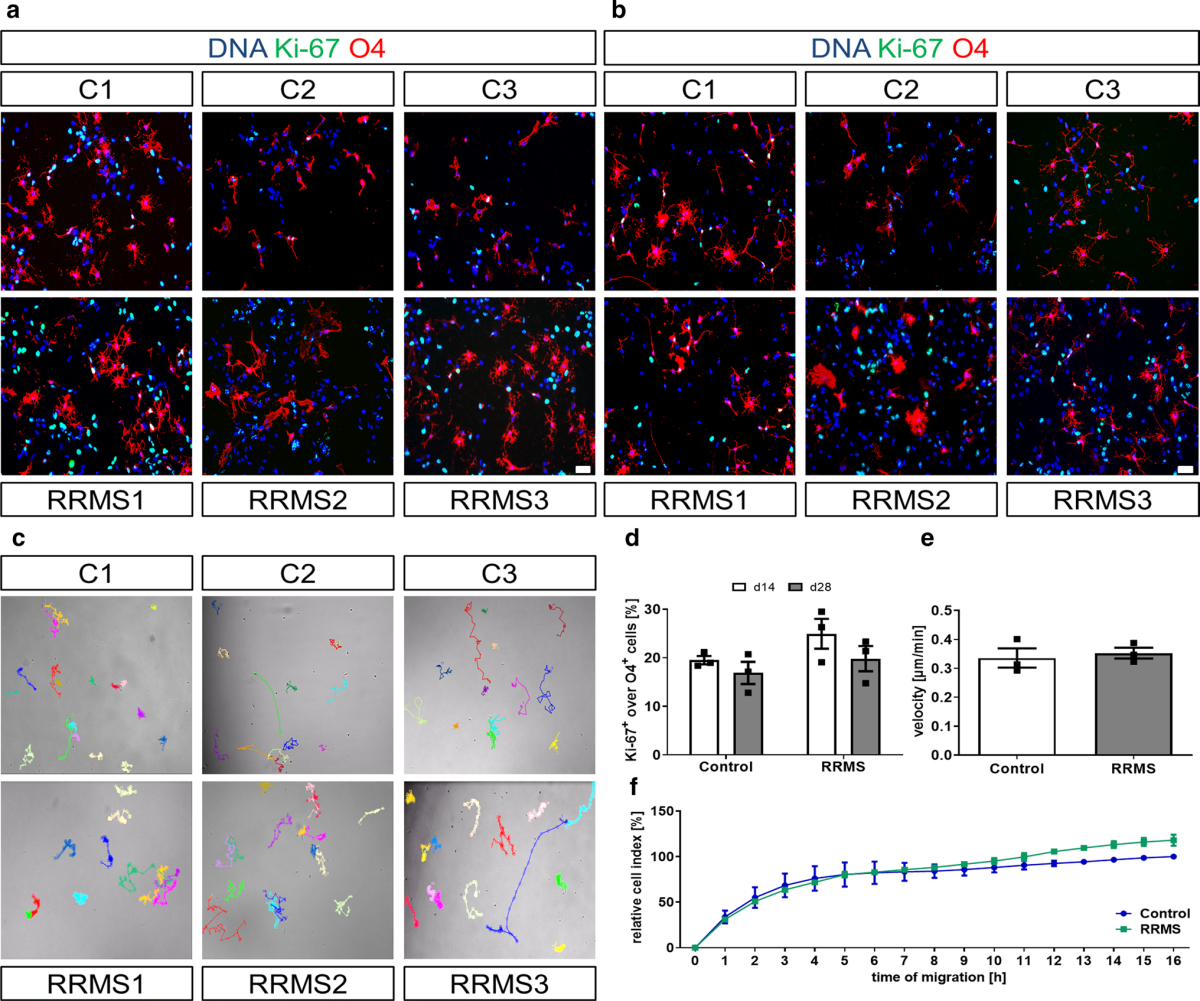
RRMS and control hiOL display similar proliferation and migration capabilities. Representative ICC of RRMS and control hiOL stained for oligodendrocyte marker O4 (red) and proliferation marker Ki-67 (green) at day 14 (**a**) and day 28 (**b**) of differentiation showing proliferating oligodendrocytes. DAPI was used to counterstain the nuclei. Scale bars: 50 µm. **c** Representative phase-contrast images of migrating O4^+^ RRMS and control hiOL. Images show the migration of O4^+^ hiOL tracked over a time period of 24 h. **d** Number of proliferating RRMS and control hiOL at day 14 and day 28 of differentiation by ICC-based quantification of Ki-67 and O4 double-positive cells showing a small decrease in proliferation at day 28 of differentiation compared to day 14 but no significant differences between RRMS and control hiOL (*n* = 3). **e** Analysis of videos recorded in **c** revealed no significant differences in velocity of migrating O4^+^ RRMS and control hiOL tracked over a time period of 24 h (*n* = 3). **f** Quantification of undirected migration of O4^+^ RRMS and control hiOL over a time period of 16 h by longitudinal impedance-based measurement confirming no significant differences in migration of RRMS and control hiOL (*n* = 3). Data are presented as mean + SEM. Statistical significance was determined by two-tailed Student’s *t* test (**d**, **e**) or Bonferroni-corrected two-way ANOVA (**f**)

To determine whether oligodendrocytes from MS patients display a disease-specific phenotype, fibroblasts from three MS patients with RRMS (RRMS) and three sex-matched healthy individuals (controls) were successfully reprogrammed into iPSCs and afterwards differentiated into NPCs (Supplementary Fig. 1, 2, online resource). To minimize genetic biases, we included fibroblasts from two monozygous twin pairs discordant for the disease in addition to one unrelated RRMS patient and one unrelated healthy individual. The non-affected twins have no signs or symptoms of MS after detailed examination by a MS specialist. Subsequently, NPCs from RRMS and control individuals were differentiated into hiOL by lentiviral transduction of SON factors. To compare the proliferation capacity, we performed double ICC for proliferation marker Ki-67 and oligodendrocyte marker O4 at day 14 and day 28 of differentiation (Fig. 1a, b). We observed a small decrease in the percentage of proliferating hiOL at day 28 compared to day 14 of differentiation, but no significant difference in the proliferation of RRMS and control hiOL (Fig. 1d). Next, we compared the migration of RRMS and control hiOL by sorting of O4^+^ cells at day 12 of differentiation and tracking of the velocity of migrating hiOL over 24 h (Fig. 1c); however, no differences were detected (Fig. 1e). This result was confirmed by the impedance-based xCELLigence system which also did not reveal any significant difference in the migration capacity of RRMS and control hiOL (Fig. 1f). In total, hiOL from RRMS patients and controls do not differ with respect to proliferation or migration.

### No differences in differentiation and ensheathment of 3D nanofibers between RRMS and control hiOL

**Fig. 2:**
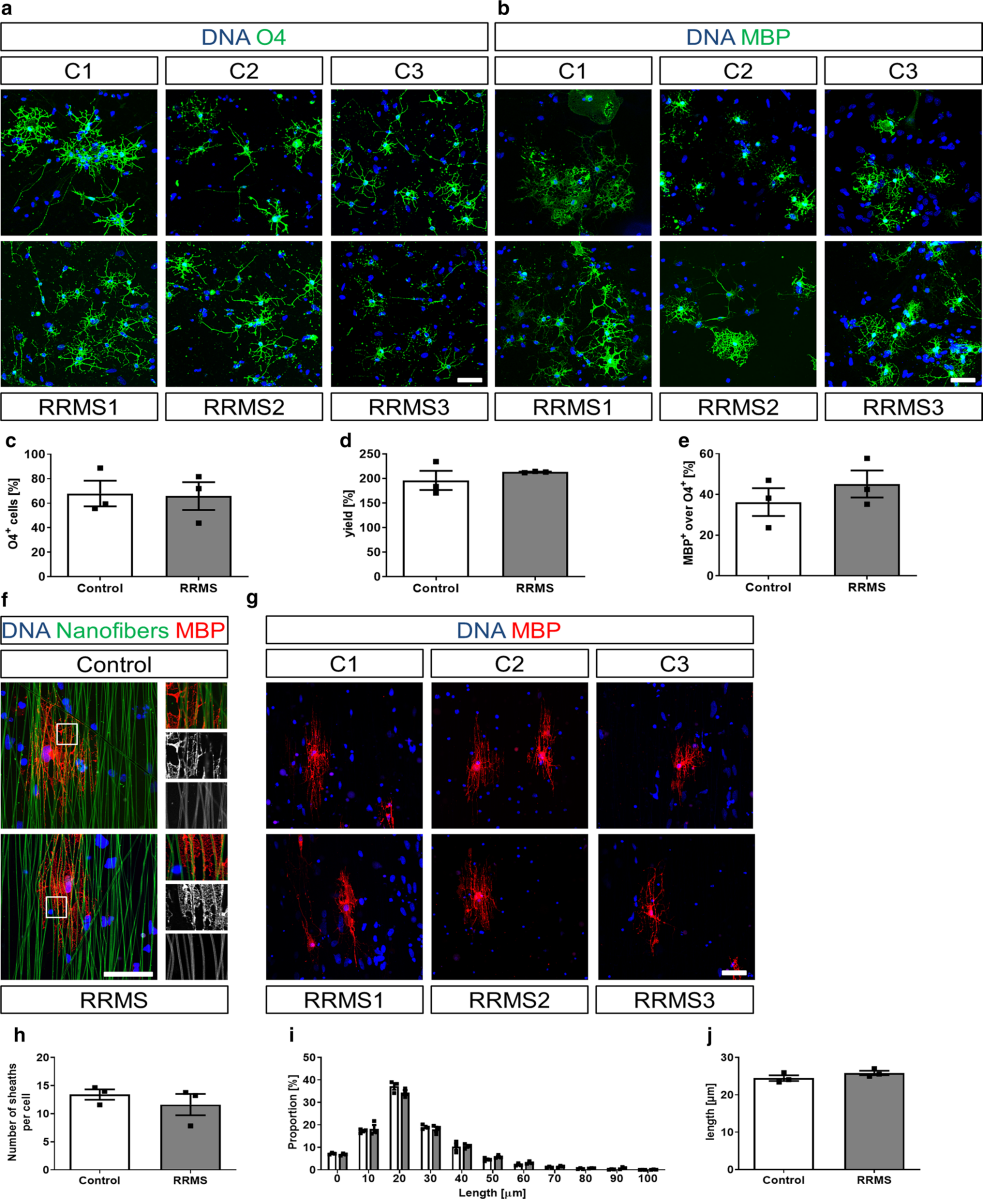
RRMS and control hiOL show the same differentiation ability and are capable of ensheathing 3D nanofibers in vitro*.* Representative ICC of RRMS and control hiOL stained for O4 (green, **a**) and MBP (green, **b**) at day 28 (**a**) or 35 (**b**) of differentiation demonstrating the differentiation of RRMS and control hiOL. Flow cytometry-based quantification at day 28 of differentiation indicating no significant differences in the percentage (**c**) and yield (**d**) of O4^+^ RRMS and control hiOL (*n* = 3). **e** Quantification of MBP^+^ hiOL over O4 at day 35 of differentiation by ICC after sorting O4^+^ hiOL by flow cytometry at day 21 displaying a similar terminal differentiation between RRMS and control hiOL (*n* = 3). **f** Representative ICC of MBP^+^ (red) hiOL of one RRMS patient and one control individual showing the formation of processes along 3D nanofibers (green). Magnification demonstrates ensheathment of nanofibers by MBP^+^ processes of RRMS and control hiOL. Boxes indicate magnified areas. **g** Representative ICC of MBP^+^ (red) RRMS and control hiOL 7 days after reseeding on 3D nanofibers showing the alignment of RRMS and control hiOL along nanofibers. Analysis of in vitro myelination capacity by heuristic algorithm indicating no differences in mean number of sheaths per cell (**h**), distribution of process lengths (**i**) or mean process length (**j**) formed by RRMS and control hiOL around 3D nanofibers (*n* = 3). Data are presented as mean + SEM. Statistical significance was determined by two-tailed Student’s *t* test (**c**–**e**, **h**, **j**) or Bonferroni-corrected two-way ANOVA (**i**). Scale bars: 50 µm; DAPI was used to counterstain the nuclei. See also Supplementary Fig. 3, online resource

Next, we examined the ability of RRMS and control NPCs to differentiate into hiOL by using O4 as a marker for immature oligodendrocytes and MBP as a marker for mature oligodendrocytes. Flow cytometry at day 28 after lentiviral transduction with SON revealed no significant difference in the percentage or the yield (amount of O4^+^ cells/amount of initially plated NPCs) of O4^+^ oligodendrocytes (Fig. 2a, c, d). MBP^+^ hiOL were quantified by ICC at day 35 after sorting of O4^+^ cells at day 21. No significant difference between the number of MBP^+^ cells over O4 between RRMS and control hiOL was found (Fig. 2b, e).

To confirm this result and rule out the possibility that overexpression of SON might obscure small differences in differentiation, we applied an in vitro patterning differentiation protocol in which oligodendroglial differentiation and maturation are achieved by growth factors and small molecules without transduction with SON (Supplementary Fig. 3a, online resource). ICC analysis confirmed that RRMS and control NPCs were able to differentiate into O4^+^ and MBP^+^ cells (Supplementary Fig. 3b, c, online resource); however, quantification of O4^+^ cells at day 23, 43, and 60 by flow cytometry did not reveal any significant difference between RRMS and control oligodendrocytes (Supplementary Fig. 3d, online resource). Since only a few MBP^+^ oligodendrocytes were observed after 60 days of differentiation (below 1%), we decided not to quantify them. Altogether, our results indicate that NPCs from RRMS patients and controls show the same capacity to differentiate into oligodendrocytes.

To analyze the myelination capacity of RRMS and control hiOL, we used an in vitro myelination assay. O4^+^ hiOL sorted at day 21 of differentiation were seeded on 3D aligned nanofibers, differentiated for an additional 7 days and stained for MBP. MBP^+^ hiOL from RRMS patients and controls extended processes, aligned to and ensheathed the nanofibers (Fig. 2f, g). Using a recently published algorithm for high-throughput quantification of oligodendroglial process formation [66], no differences between RRMS and control hiOL were observed with respect to the mean number of sheaths per cell, the mean length of the processes per cell or the distribution of process lengths (Fig. 2h–j) demonstrating that RRMS and control hiOL do not differ in their capability to form MBP^+^ sheaths along 3D structures.

### RRMS and control hiOL display no difference in stress response

To analyze the stress response, we applied different stressors to RRMS and control hiOL. O4^+^ RRMS and control hiOL sorted at day 21 of differentiation were exposed to different concentrations of the respiratory chain inhibitor rotenone and reactive oxygen producer peroxynitrite for 24 h at day 25 of differentiation. Furthermore, we tested the effect of the MS-associated cytokines tumor necrosis factor-α (TNFα) and IFNγ on oligodendroglial viability for 48 h. Rotenone and peroxynitrite, in contrast to TNFα and IFNγ, induced a significant concentration-dependent decrease in viability; however, RRMS and control hiOL were affected in a comparable manner (Supplementary Fig. 4a–d, online resource). These data suggest that hiOL from RRMS patients and controls have the same susceptibility to oxidative stress.

### RRMS and control hiOL display a similar proteome

**Fig. 3:**
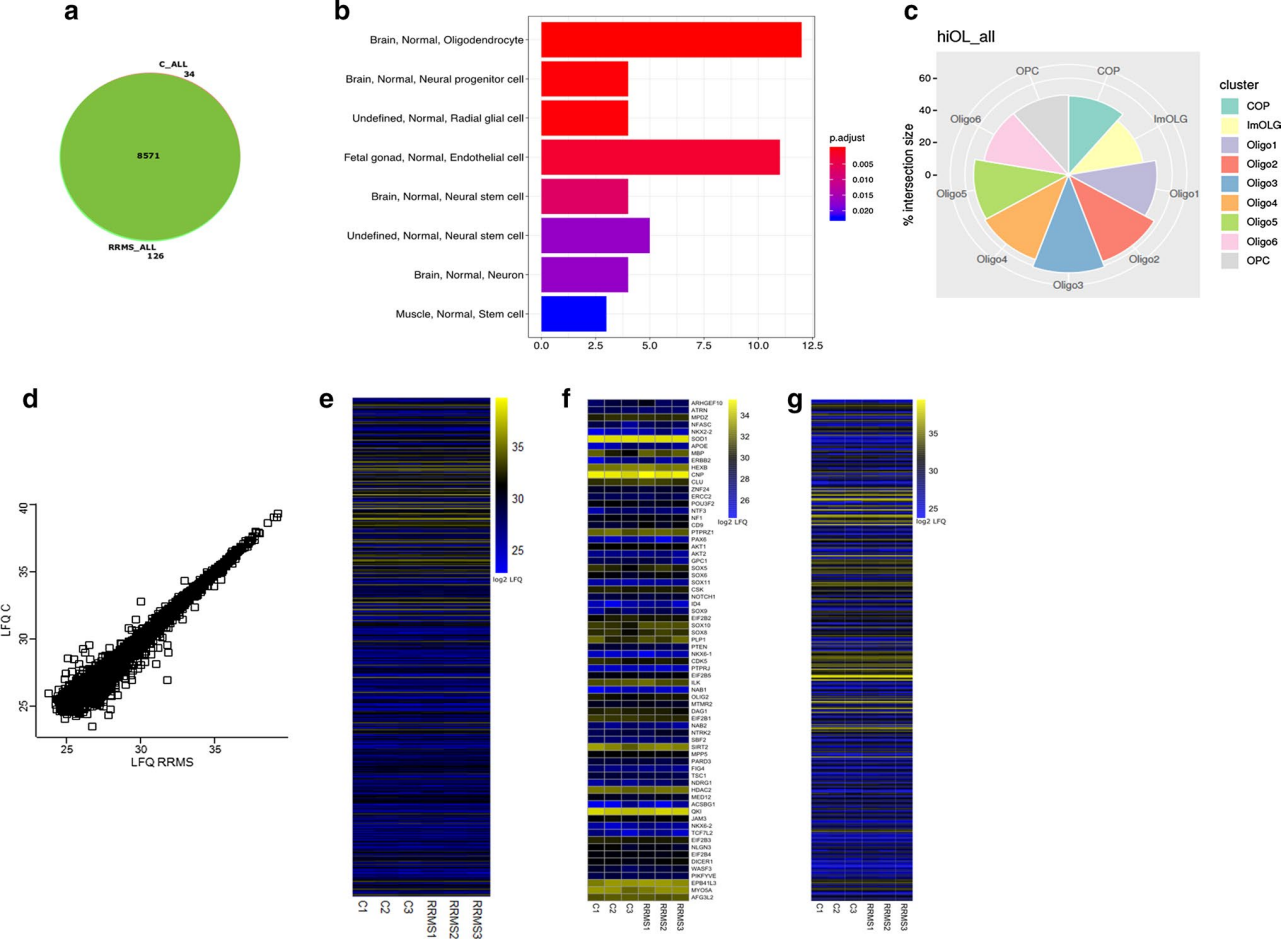
RRMS and control hiOL display highly similar proteomes. **a** Venn-Diagram showing proteins detected only in pooled control hiOL (C), pooled RRMS hiOL (RRMS) or both indicating that the majority of proteins is shared between both groups. **b** Cell marker enrichment analysis of proteins with mean log2 transformed LFQ intensities > 35 across the whole data set demonstrating that proteins characteristic for oligodendrocytes are significantly enriched in the data set. **c** Polarplot showing the intersection of all proteins with markers characterizing oligodendroglial subclusters identified with single-nucleus RNA sequencing described in Jäkel et al. [23]. Circles indicate percentages of the intersection with distinct clusters. **d** Scatter plot of log2 transformed LFQ intensities of control and RRMS hiOL indicating a strong correlation between the proteomes of RRMS and control hiOL. **e** HeatMap of all identified proteins based on log2 transformed LFQ intensities indicating that protein expression is highly similar between RRMS and control hiOL. **f** HeatMap of log2 transformed LFQ intensities for proteins associated with GO terms “central nervous system myelination”, “myelin maintenance”, “myelin assembly”, “myelination”, “oligodendrocyte cell fate specification”, “oligodendrocyte development”, “oligodendrocyte differentiation”, and “positive regulation of oligodendrocyte differentiation” demonstrating presence and similar expression of oligodendroglial proteins in all analyzed samples. **g** HeatMap of log2 transformed LFQ intensities for proteins associated with GO term “immune response” indicating the presence and similar expression levels of proteins connected to the immune system in all analyzed samples. Proteomic analysis was performed in two independent experiments with three RRMS patients and three healthy controls including in total two different NPC clones per patient and healthy control. Statistical significance was determined by Student’s *t*-test with permutation-based FDR. See also Supplementary Fig. 5, online resource

To further characterize and compare the cellular identity of RRMS and control hiOL we analyzed the proteome. To this end, O4^+^ RRMS and control hiOL sorted at day 28 of differentiation were analyzed by quantitative label-free LC–MS/MS to detect and identify differentially regulated proteins. In total, we could identify 8731 protein groups with the vast majority (8571) being shared between control and RRMS hiOL (Fig. 3a). Analysis of proteins with mean log2 transformed LFQ intensities > 35 across RRMS and control hiOL revealed that proteins specific for oligodendrocytes were significantly enriched in the data set (Fig. 3b), thus confirming the cellular identity of hiOL. Moreover, we searched for proteins encoded by genes recently described in oligodendroglial subclusters identified by single-nucleus RNA sequencing [23]. Indeed, we identified markers for every cluster on protein level (Fig. 3c). Comparison of the pooled proteomes of RRMS and control hiOL indicates a strong correlation between control and RRMS hiOL samples (Pearson-correlation coefficient 0.998) (Fig. 3d) suggesting that overall differences in protein expression levels between both cell types are rather marginal. Applying a Student’s *t*-test with permutation-based FDR to correct for multiple hypotheses testing to the data set indeed confirmed the absence of any significantly regulated protein. In addition, hierarchical clustering of log2 transformed LFQ intensities of all detected proteins demonstrated very similar intensity distributions across all samples (Fig. 3e). In addition, GO terms referring to proteins involved in “oligodendrocyte development”, “oligodendrocyte differentiation”, “positive regulation of oligodendrocyte differentiation”, “central nervous system myelination”, “myelin assembly”, “myelin maintenance”, and “myelination” were not significantly altered (Fig. 3f). Since the role of oligodendrocytes in immune responses is currently highly debated and we could identify markers referring to the subcluster “immune oligodendroglia (ImOLG)” described in Jäkel et al. [23], GO term analysis was used to analyze proteins associated with immune responses (Supplementary Table 5, online resource). Indeed, we could identify a high number of proteins related to the GO term “immune response” (e.g. CD74, CYFIP1, and ITPR2), however, the log2 transformed LFQ intensities were similarly distributed between RRMS and control hiOL (Fig. 3g). In summary, these data demonstrate that the proteomes of RRMS and control hiOL display subcluster specific markers identified in earlier studies [23] and are highly similar.

To validate these results and exclude that overexpression of SON results in a shift of the proteomic composition, we repeated LC–MS/MS measurements with iPSC-derived oligodendrocytes that were differentiated from NPCs by an in vitro patterning approach achieved without overexpression of SON (Supplementary Fig. 5, online resource). We identified 7740 protein groups and the vast majority was shared between control and RRMS iPSC-derived oligodendrocytes (Supplementary Fig. 5a, online resource). Analysis of the proteins whose LFQ intensities account for the top 10% of all LFQ intensities revealed that also when utilizing the in vitro patterning approach, proteins specific for oligodendrocytes were significantly enriched in the data set (Supplementary Fig. 5b). Similar to the results obtained with overexpression of SON, we found a strong correlation between control and RRMS samples (Pearson-correlation coefficient 0.979) (Supplementary Fig. 5c, online resource), which was underlined by applying a Student’s *t* test with permutation-based FDR to correct for multiple hypotheses testing only identifying a single differently regulated protein [Guanine nucleotide-binding protein G(I)/G(S)/G(O) subunit gamma-4 (GNG4)]. Again, hierarchical clustering of log2 transformed LFQ intensities of all detected proteins demonstrated very similar intensity distributions across all samples and neither GO terms referring to proteins involved in “oligodendrocyte development”, “oligodendrocyte differentiation”, “positive regulation of oligodendrocyte differentiation”, “central nervous system myelination”, “myelin assembly”, “myelin maintenance”, and “myelination” nor GO terms associated with “immune response” (Supplementary Table 6, online resource) were significantly altered (Supplementary Fig. 5d–f, online resource). Combined, these data confirm a high similarity between the proteomes of RRMS and control iPSC-derived oligodendrocytes.

### Supernatants of activated PBMCs impair the differentiation of hiOL

**Fig. 4:**
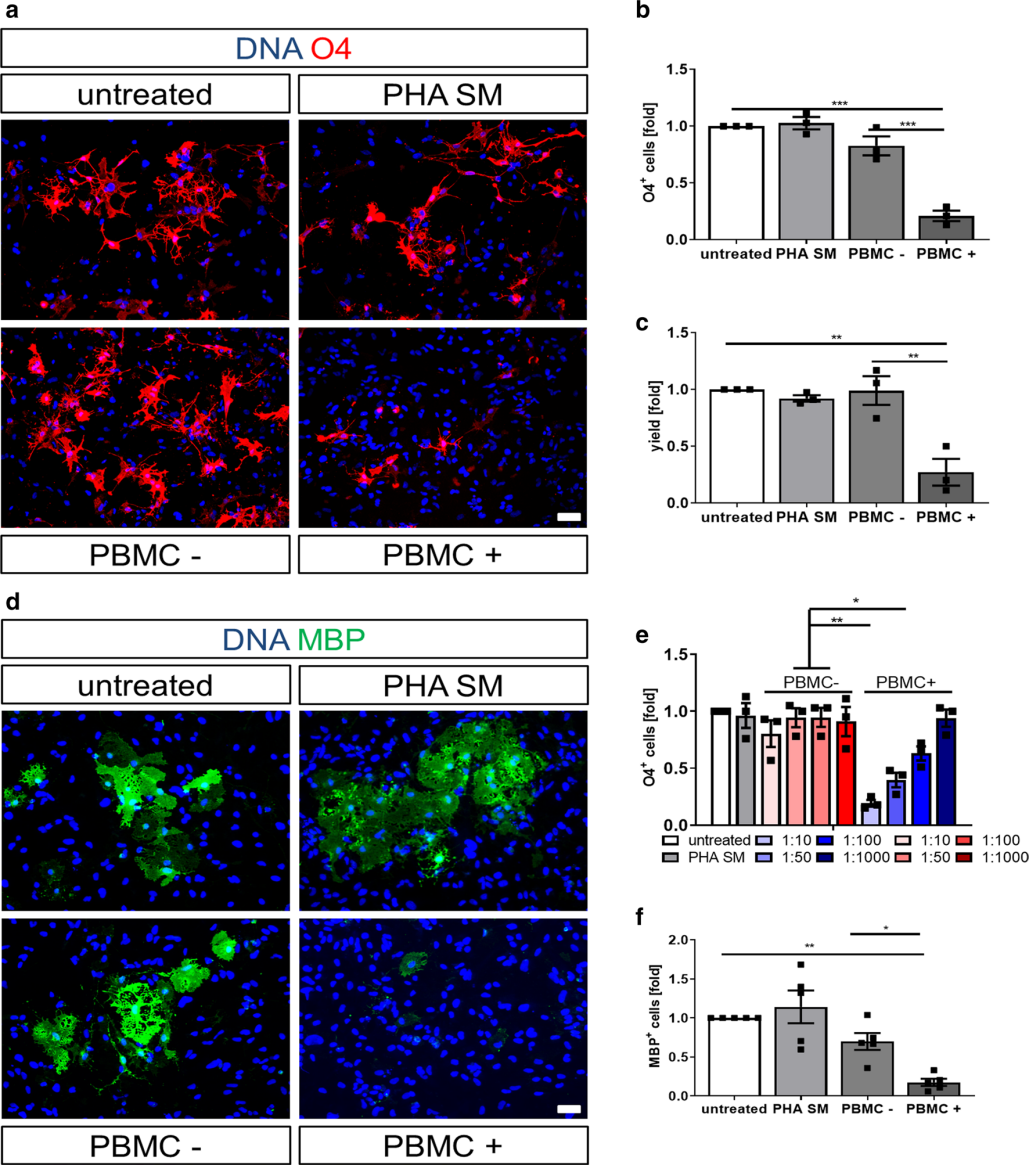
Supernatants of activated PBMCs significantly impair the differentiation into hiOL. **a** Representative ICC of O4^+^ (red) hiOL of healthy donors that were either left untreated or treated with PHA-containing stimulation medium (PHA SM) or the supernatants of (non-) activated PBMCs (PBMC–/+) from day 4 until day 21. Flow cytometry-based quantification of O4^+^ hiOL at day 21 of differentiation indicating a significantly decreased percentage (**b**) and yield (**c**) of O4^+^ hiOL by PBMC+ (*n* = 3). **d** Representative ICC of MBP^+^ (green) hiOL at day 35 of differentiation. Untreated cells were sorted by flow cytometry for O4 at day 21 of differentiation and subsequently either left untreated or treated with PHA SM, PBMC−, or PBMC+ until day 35. **e** Flow cytometry-based quantification of O4^+^ hiOL treated with different dilutions of PBMC supernatants indicating a dose-dependent impaired differentiation into hiOL by PBMC+ at day 21 of differentiation (*n* = 3). **f** Quantification of MBP^+^ hiOL at day 35 of differentiation by ICC after sorting of untreated O4^+^ hiOL by flow cytometry at day 21 displaying an impaired differentiation into MBP after treatment with PBMC+ (*n* = 5). Data are presented as mean + SEM. Statistical significance was determined by Bonferroni-corrected one-way ANOVA (*p < 0.05, **p < 0.01, ***p < 0.001). The relative number of O4/MBP^+^ cells present under untreated conditions (**b**, **c**, **e**, **f**) was arbitrarily set to 1 and used to normalize in a pairwise fashion. Scale bars: 50 µm; DAPI was used to counterstain the nuclei. See also Supplementary Fig. 6–9, online resource.

Since MS lesions are characterized by a variety of immune cells invading the CNS from the periphery, we next asked whether the inflammatory milieu itself may impair the differentiation of OPCs into mature oligodendrocytes in MS lesions. To test this hypothesis, we applied the supernatants of PBMCs which consist of a variety of different immune cells from the peripheral blood, including T cells, B cells, and monocytes (Supplementary Fig. 6f, online resource) to differentiating hiOL from healthy individuals. Polyclonal stimulation of PBMCs with PHA induced the upregulation of cellular activation markers such as CD69 and HLA-DR, hence confirming the stimulatory effect of PHA in our setup (Supplementary Fig. 6a, online resource). Notably, incubation of hiOL with supernatants of activated PBMCs (PBMC+) significantly impaired the differentiation into O4^+^ cells compared to supernatants of non-activated PBMCs (PBMC–), PHA-containing stimulation medium (PHA SM) or the untreated control (Fig. 4a, b). This result was confirmed by quantification upon culture with increasing concentrations of PBMC supernatants by flow cytometry showing significantly decreased numbers of O4^+^ hiOL at day 21 of differentiation in a dose-dependent manner (Fig. 4e). Additionally, the yield of O4^+^ cells was significantly reduced after application of PBMC+ at day 21 of differentiation (Fig. 4c). To investigate the effect of PBMC+ on the terminal differentiation of hiOL, we sorted untreated O4^+^ cells at day 21 of differentiation and differentiated the cells for an additional 14 days in the presence of PBMC supernatants. The quantification of MBP^+^ cells revealed a significantly impaired terminal differentiation (Fig. 4d, f).

To see whether RRMS hiOL are affected in a comparable manner by PBMC+ , we analyzed early and terminal differentiation of RRMS hiOL after application of PBMC supernatants. We found that early and terminal hiOL differentiation of RRMS patients were reduced by PBMC+ in a comparable manner as in controls (Supplementary Fig. 7, online resource). Based on these results, we used only hiOL of healthy donors for the following experiments.

To exclude that the reduced presence of O4^+^ and MBP^+^ cells is due to increased cell death, we quantified the total number of cells after 21 days of differentiation (Supplementary Fig. 8a, online resource) and performed flow cytometry analysis for Annexin V and the dead cell marker PI (Supplementary Fig. 8b, c, online resource). PBMC+ did not reduce cell numbers or increased cell death in any of the assays but even reduced the percentage of dead cells (Supplementary Fig. 8c, online resource). This confirms that PBMC+ impair oligodendroglial differentiation, but do not induce cell death.

To examine whether the effect of PBMC+ is specific for hiOL, we exposed NPCs differentiating into neurons to PBMC supernatants. The application of PBMC+ did not significantly affect the differentiation into TUJ1^+^ neurons (Supplementary Fig. 9a, b, online resource), revealing that PBMC+ specifically inhibit the differentiation into hiOL.

### T cells are responsible for impaired oligodendroglial differentiation and the number of T cells correlates inversely with the extent of remyelination in MS patients

**Fig. 5:**
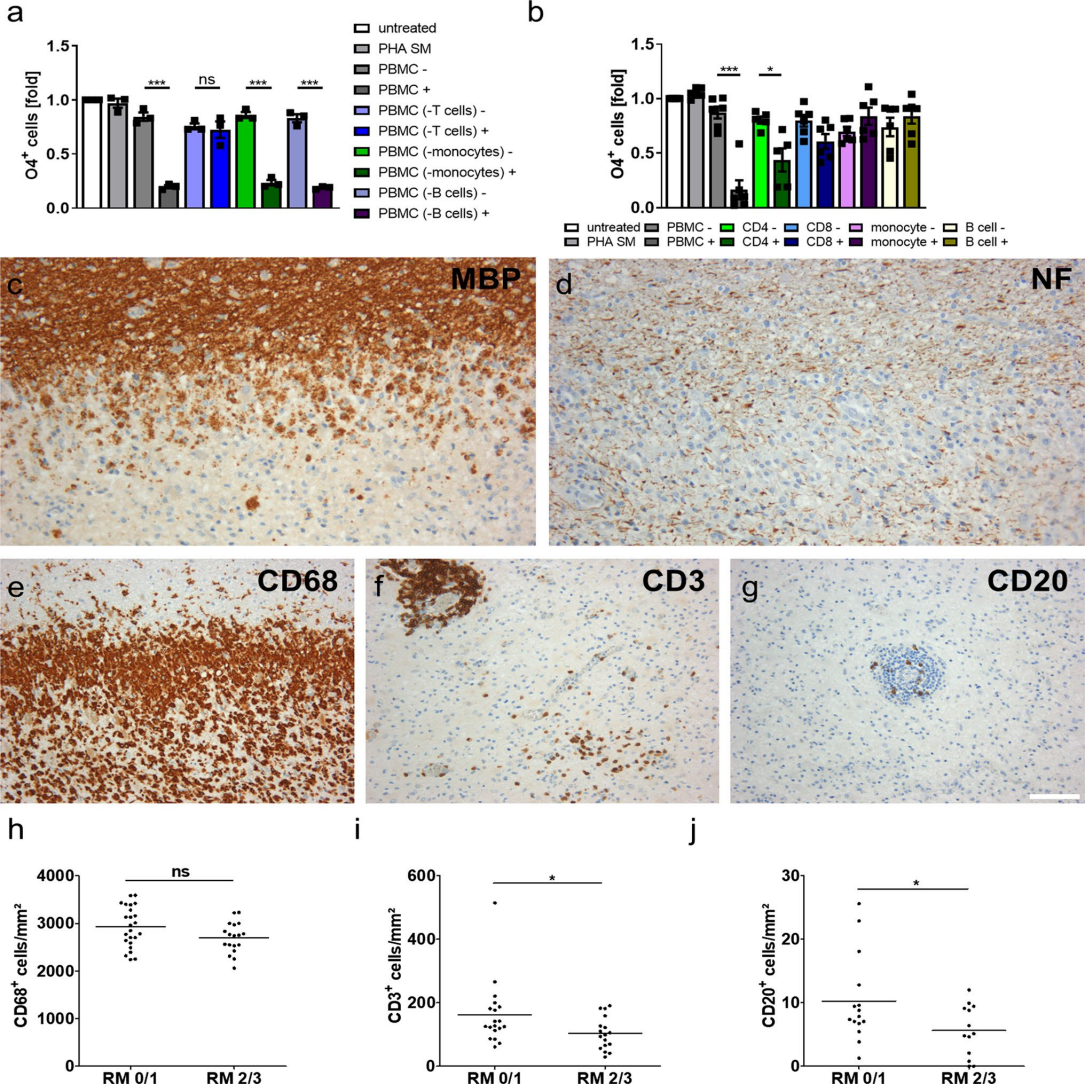
T cells mediate the impaired oligodendroglial differentiation and correlate negatively with remyelination in MS patients. **a** Flow cytometry-based quantification of O4^+^ hiOL at day 21 of differentiation. Cells were either left untreated or treated with PHA SM, PBMC−/+, supernatants of (non-) activated monocyte-depleted PBMCs (PBMC (-monocytes)−/+), supernatants of (non-) activated B cell-depleted PBMCs (PBMC (-B-cells)−/+), or supernatants of (non-) activated T cell-depleted PBMCs (PBMC (-T-cells)−/+). Depletion of T cells completely restores differentiation ability of hiOL (*n* = 3). **b** Flow cytometry-based quantification of O4^+^ hiOL at day 21 of differentiation. Cells were either left untreated or treated with PHA SM, PBMC−/+, supernatants of (non-) activated CD4^+^ T cells (CD4−/+), supernatants of (non-) activated CD8^+^ T cells (CD8−/+), supernatants of (non-) activated CD14^+^ monocytes (CD14−/+), or supernatants of (non-) activated CD19^+^ B cells (CD19−/+). CD4+ significantly inhibit the differentiation of hiOL (*n* = 6). Representative IHC of an active MS lesion showing demyelination and relative preservation of axons indicated by MBP (**c**) and neurofilament (NF) (**d**) staining. Infiltration of numerous CD68^+^ macrophages/microglia (**e**) characterizes active MS lesions. Additionally, CD3^+^ T cells (**f**) and CD20^+^ B cells (**g**) are present within the lesions. Analysis of the extent of remyelination (RM) in association with number of CD68^+^ macrophages/microglia (**h**) (*n* = 42), CD3^+^ T cells (**i**) (*n* = 36), and CD20^+^ B cells (**j**) (*n* = 28) demonstrating a correlation between impaired remyelination (RM 0/1) and increased numbers of T cells and B cells but not macrophages/microglia. Data are presented as mean + SEM (**a**, **b**). Statistical significance was determined by Bonferroni-corrected one-way ANOVA (**a**, **b**) or Student’s *t* test (**h**–**j**) (*ns* not significant, *p < 0.05, **p < 0.01, ***p < 0.001). The relative number of O4^+^ cells (**a**, **b**) present under untreated conditions was arbitrarily set to 1 and used to normalize in a pairwise fashion. Scale bar: 100 µm. See also Supplementary Fig. 10, online resource

To identify the cell type in PBMCs mediating impaired differentiation of hiOL, we next depleted distinct immune cell populations from PBMCs (Supplementary Fig. 6g–j, online resource) and compared the effects of the respective supernatants. Neither depletion of B cells nor monocytes from PBMCs could rescue impaired differentiation upon addition of PBMC+, whereas depletion of T cells completely restored the differentiation into O4^+^ hiOL (Fig. 5a). To reciprocally investigate whether T cells alone are capable to impair oligodendrocyte differentiation, we next applied supernatants from isolated immune cell populations upon stimulation to differentiating hiOL (Supplementary Fig. 6b–e, online resource). In line with the results from the depletion experiments, supernatants from isolated and stimulated B cells and monocytes did not affect oligodendroglial differentiation. Interestingly, supernatants of activated CD4^+^ T cells (CD4+) but not supernatants of activated CD8^+^ T cells significantly impaired hiOL differentiation (Fig. 5b) indicating that the oligodendroglial differentiation block is predominantly caused by factors produced by CD4^+^ T cells. To show reproducibility of this effect, PBMC+ and CD4+ from five different donors were applied to differentiating cells. Impaired differentiation into O4^+^ hiOL was observed for all five donor supernatants (Supplementary Fig. 10a–e, online resource).

To determine the contribution of distinct immune cell types on remyelination in MS, we next analyzed the abundance of CD68^+^ macrophages/microglia, CD3^+^ T cells, and CD20^+^ B cells in active MS lesions since this lesion type is prone to remyelination [53]. Therefore, we investigated biopsies from 32 MS patients and identified lesions based on loss of myelin and relative preservation of axons (Fig. 5c, d). Active lesions are characterized by a diffuse infiltration with numerous CD68^+^ macrophages/microglia throughout the complete lesion area (Fig. 5e) [30]. As expected, macrophages/microglia were the most abundant immune cell type in active MS lesions (Fig. 5e, h). Additionally, we observed high numbers of T cells (Fig. 5f, i) but very few B cells (Fig. 5g, j). Notably, the number of T cells and B cells but not the number of macrophages/microglia correlated inversely with the extent of remyelination assessed using a semiquantitative score [18] (Fig. 5h–j). In summary, these in vitro and in vivo data suggest a significant contribution of T cells to impaired remyelination in the CNS.

### IFNγ secreted by PBMCs impairs differentiation of hiOL

**Fig. 6:**
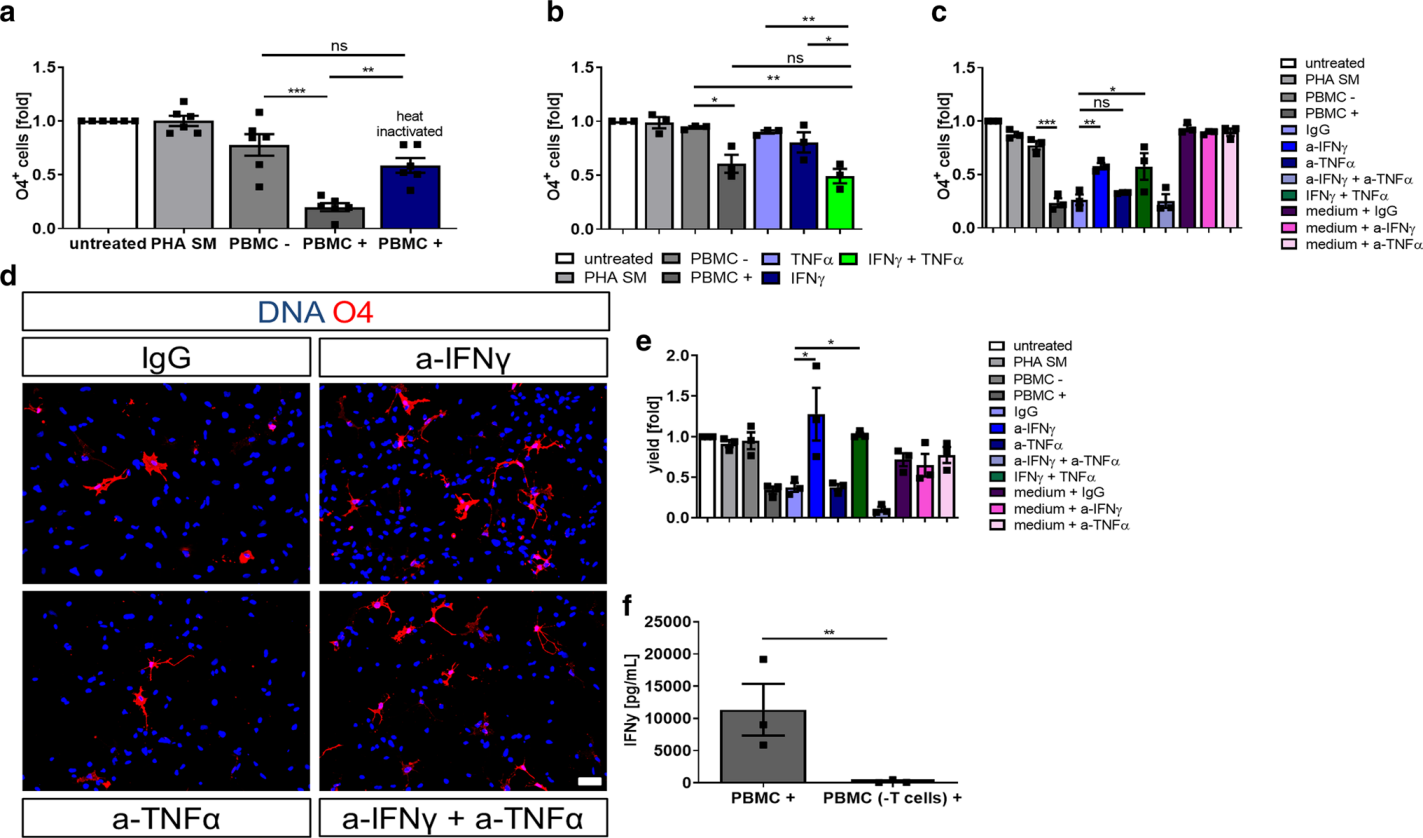
Neutralization of IFNγ but not TNFα restores the differentiation into hiOL. **a** Flow cytometry-based quantification of O4^+^ hiOL at day 21 of differentiation. Cells were either left untreated or treated with PHA SM, PBMC−/+, or heat-inactivated PBMC+. Heat inactivation of PBMC+ restores the differentiation into O4^+^ hiOL (*n* = 6). **b** Flow cytometry-based quantification of O4^+^ hiOL at day 21 of differentiation while being treated with IFNγ (400 ng/mL), TNFα (200 ng/mL), or combination of both showing a significantly impaired differentiation into hiOL when a combination of IFNy and TNFα was applied (*n* = 3). Flow cytometry-based quantification of O4^+^ hiOL at day 21 of differentiation indicating a significantly enhanced percentage (**c**) and yield (**e**) of O4^+^ hiOL when PBMC+ were treated with a-IFNy before compared to IgG (*n* = 3). **d** Representative ICC of O4^+^ (red) hiOL that were treated with PBMC+ which were either incubated with IgG, a-IFNy, a-TNFα, or a-TNFα+ a-IFNy beforehand. Supernatants were applied from day 4 until day 21 and staining was performed at day 21. **f** ELISA-based analysis of IFNγ in PBMC+ and PBMC (-T cells)+ showing significantly reduced concentrations of IFNγ after depletion of T cells from PBMC+ (*n* = 3). Data are presented as mean + SEM. Statistical significance was determined by Bonferroni-corrected one-way ANOVA (**a**–**c**, **e**) or two-tailed Student’s *t* test (**f**) (*ns* not significant, *p < 0.05, **p < 0.01, ***p < 0.001). The relative number of O4^+^ cells (**a**–**c**, **e**) present under untreated conditions was arbitrarily set to 1 and used to normalize in a pairwise fashion. Scale bar: 50 µm. DAPI was used to counterstain the nuclei. See also Supplementary Figs. 11, 12, online resource

To elucidate which factors in PBMC+ are responsible for the impaired hiOL differentiation, we heat-inactivated PBMC+. We observed restored differentiation into O4^+^ hiOL suggesting that the impaired differentiation of hiOL is caused by heat-susceptible factors, such as secreted proteins (Fig. 6a).

Since PBMCs and especially T cells produce high levels of proinflammatory cytokines such as TNFα and IFNγ (Supplementary Fig. 11a, b, online resource), we determined the effects of these two cytokines on oligodendroglial differentiation by exposing differentiating hiOL to recombinant TNFα and IFNγ either alone or in combination. Whereas neither the application of TNFα nor IFNγ alone affected hiOL differentiation, combined application of both cytokines led to a significantly impaired differentiation into hiOL (Fig. 6b). Neutralizing TNFα and IFNγ antibodies resulted in a reduction of TNFα and IFNγ levels in PBMC+ compared to IgG controls (Supplementary Fig. 11c, d, online resource). Neutralizing IFNγ either alone or in addition to TNFα restored the differentiation into O4^+^ hiOL, whereas blocking of TNFα alone had no effect on impaired hiOL differentiation induced by PBMC+ (Fig. 6c, d). Moreover, yields of O4^+^ cells were significantly increased after blocking IFNγ or combination of IFNγ and TNFα (Fig. 6e). To determine whether T cells are responsible for IFNγ secretion in PBMC supernatants, we analyzed IFNγ levels in PBMC+ and T cell-depleted PBMC+ (PBMC (−T cells)+) of three different donors. Whereas PBMC+ contained large amounts of IFNγ, IFNγ secretion was significantly reduced in PBMC (−T cells)+ (Fig. 6f) indicating that T cells are essential for IFNγ secretion in PBMCs.

As it was recently described that IFNγ induces the expression of immune markers on rodent oligodendrocytes which suggests a putative role for oligodendrocytes in modulating immune responses [15, 27], we next analyzed whether this also applies to human oligodendrocytes. To this end, we treated differentiating hiOL with PBMC+, PBMC+ in which IFNγ was neutralized, recombinant IFNγ and the combination of recombinant IFNγ and TNFα. We found that PBMC+ as well as IFNγ and TNFα induced the expression of MHC-I, MHC-II and immune adhesion molecules on hiOL on a transcriptional (Supplementary Fig. 12a–e, online resource) and translational level (Supplementary Fig. 12f–j, online resource). Notably, neutralization of IFNγ from PBMC+ reduced enhanced transcript and protein levels. Combined, these data indicate the expression of immune markers by hiOL in an inflammatory environment which is at least partly mediated by secreted IFNγ.

### Immunomodulatory treatment of PBMC partly restores impaired hiOL differentiation mediated by PBMC+ 

**Fig. 7:**
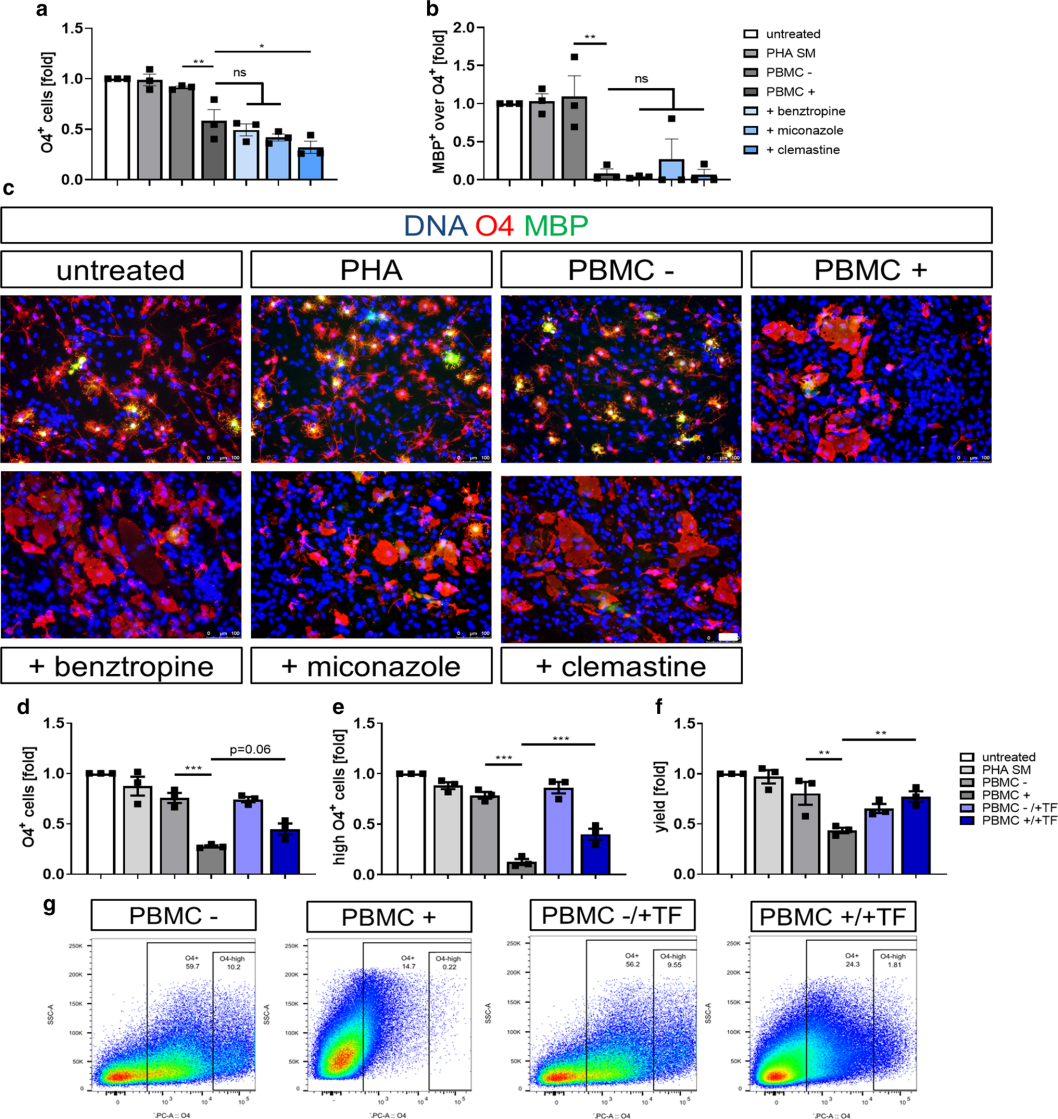
Immunomodulatory treatment of PBMCs can partly restore impaired hiOL differentiation induced by supernatants of activated PBMCs. **a** Flow-cytometry based quantification of O4^+^ hiOL at day 21 of differentiation demonstrating that differentiation-promoting drugs benztropine, miconazole, and clemastine are not able to restore hiOL differentiation under application of PBMC+ (*n* = 3). **b** Quantification of MBP^+^ over O4^+^ hiOL at day 35 of differentiation by ICC after sorting of untreated O4^+^ hiOL by flow cytometry at day 21 displaying that differentiation-promoting drugs benztropine, miconazole, and clemastine are not able to restore impaired hiOL differentiation induced by PBMC+ (*n* = 3). **c** Representative ICC of MBP^+^ (green) over O4^+^ (red) hiOL at day 35 of differentiation. Untreated cells were sorted by flow cytometry for O4 at day 21 of differentiation and subsequently either left untreated or treated with PHA SM, PBMC−/+, or PBMC+ plus differentiation-promoting drugs until day 35. Flow cytometry-based quantification of O4^+^ hiOL (**d**, **f**) or high-O4^+^ hiOL (**e**) at day 21 of differentiation. Percentage of high-O4^+^ hiOL and yield of O4^+^ hiOL are significantly enhanced when PBMCs from three different donors were treated with TF prior to collection of supernatants, while percentage of total O4^+^ cells shows a trend to increased differentiation (*n* = 3). **g** Representative flow cytometry-plots at day 21 of differentiation demonstrating the distribution of O4^+^ and the subgroup high-O4^+^ cells after addition of PBMC−, PBMC+ , PBMC−/+ TF, or PBMC+/+ TF to differentiating hiOL. Data are presented as mean + SEM. Statistical significance was determined by Bonferroni-corrected one-way ANOVA (*ns* not significant, *p < 0.05, **p < 0.01, ***p < 0.001). The relative number of O4^+^/MBP^+^ over O4^+^ cells (**a**, **b**, **d**–**f**) present under untreated conditions was arbitrarily set to 1 and used to normalize in pairwise fashion. Scale bar: 50 µm. DAPI was used to counterstain the nuclei. See also Supplementary Fig. 13, online resource

Next, to determine whether impaired hiOL differentiation after application of PBMC+ can be restored by addition of drugs which have been shown to promote oligodendroglial differentiation [12, 14, 41, 42], we applied benztropine, miconazole, and clemastine simultaneously with PBMC+ to differentiating hiOL. Different concentrations of miconazole and benztropine have been shown to promote early and terminal hiOL differentiation in a previous study from our group and we used the most effective concentration identified in this study for our current experiments [14]. We additionally included clemastine although we did not observe a significantly enhanced hiOL differentiation previously [14]. However, it promoted oligodenroglial differentiation in other studies and has been used in clinical trials [19, 41]. Furthermore, the molecular mechanisms required to promote endogenous oligodendroglial differentiation may differ from the mechanisms that promote oligodendroglial differentiation in an inflammatory context. However, none of these drugs was able to rescue impaired early or terminal oligodendroglial differentiation induced by PBMC+ (Fig. 7a–c).

To elucidate whether instead an immunomodulatory treatment of PBMCs before collection of supernatants can restore the differentiation of hiOL, we treated PBMCs from 3 different donors with the immunomodulatory drug teriflunomide (TF) during activation with PHA. Treatment of PBMCs with TF which inhibits the proliferation of immune cells [4] resulted in a significantly reduced but not completely abolished IFNγ secretion during activation (Supplementary Fig. 13, online resource). There was a trend for, but not a significant increase in the percentage of total O4^+^ cells (Fig. 7d). Furthermore, treatment of PBMCs with TF increased significantly the percentage of strongly O4^+^ hiOL suggesting an enhanced differentiation in a subpopulation of oligodendrocytes (Fig. 7e and g). Additionally, administration of PBMC+/+TF resulted in significantly enhanced O4^+^ yields (amount of O4^+^ cells/amount of initially plated NPCs) (Fig. 7f).

In summary, these data demonstrate that immune cell-derived inflammatory factors such as IFNγ can directly impair oligodendroglial differentiation. Treatment of oligodendrocytes with compounds promoting oligodendroglial differentiation was not able to rescue impaired oligodendroglial differentiation induced by PBMC . However, treatment of PBMCs with the immunomodulatory drug TF before supernatant collection showed a significant effect on the oligodendroglial yield as well as on the differentiation of an oligodendroglial subpopulation suggesting that immunomodulatory drugs might have the potential to increase oligodendroglial differentiation via modulation of the inflammatory environment.

## Discussion

Remyelination failure in MS has been attributed to impaired differentiation of OPCs towards mature oligodendrocytes. Prerequisite for successful remyelination in MS is the proliferation and migration of OPCs as well as their differentiation into myelin-forming mature oligodendrocytes. All of these different steps might be impaired in MS lesions; however, impaired oligodendroglial differentiation appears to be a major contributor to remyelination failure in MS [8, 29, 63, 64]. Here, we demonstrate that hiOL from RRMS patients and controls do not differ in their functionality or proteome; however, inflammatory mediators and especially IFNγ, released by T cells, inhibit oligodendroglial differentiation which cannot be restored by the application of oligodendroglial differentiation promoting drugs but can partly be enhanced by imunomodulatory treatment of PBMCs suggesting that the inflammatory environment in MS lesions is a major contributor to impaired remyelination in MS.

During recent years there is a continuing debate over whether primary oligodendroglial pathology might contribute to lesion development and remyelination failure in MS. This hypothesis has been based on the identification of patients with significant oligodendroglial cell death in newly forming MS lesions interpreted as a primary oligodendroglial pathology resulting in secondary inflammation and demyelination [3, 38], the presence of reduced myelin and axonal densities despite lack of inflammation [50], and the description of subgroups of MS patients with either extensive or limited remyelination [46, 47]. Our study is to our best knowledge the first one analyzing in detail functions of human oligodendrocytes required for successful remyelination. Our results suggest that there are no major functional differences between the oligodendrocytes from RRMS patients and controls. This is in line with earlier observations reporting the formation of compact myelin sheaths after transplantation of O4^+^ iPSC-derived OPCs from four primary progressive MS (PPMS) patients into immunodeficient shiverer mice and the comparable differentiation of iPSC-derived oligodendrocytes from two PPMS patients and two controls into O4^+^ and MBP^+^ oligodendrocytes in vitro [13, 17]. Although the number of patients investigated in these individual studies is low, a caveat immanent to studies using patient-derived iPSCs, they all point in a similar direction, namely that a primary MS-specific oligodendroglial phenotype is not a major contributor to disease pathogenesis in MS. However, a recently published study described increased levels of some cellular senescence markers in NPCs derived from iPSCs from PPMS patients compared to control NPCs. Treatment with rapamycin reduced cellular senescence and restored the capability of NPC supernatants to promote differentiation of rodent oligodendrocytes [43]. These results might indicate that age-specific changes contribute in a disease-specific manner to disease pathology in PPMS.

Recently, using single-nucleus RNA sequencing disease-specific oligodendroglial clusters have been identified in MS patients as well as in experimental autoimmune encephalomyelitis (EAE) mice which serve as an animal model for MS [15, 23], however, the expression of the majority of markers for distinct clusters has not been confirmed on protein level yet. hiOL express all major myelin-associated proteins, such as CNP, PLP, MBP, MAG, MOG etc., confirming the oligodendroglial identity, but also some of the proteins which have been found to be unique or enriched on the transcriptional level in oligodendroglial subclusters identified in MS and control brains, such as PDGFRα, BCAN, SOX6, APOE and CD74 [23]. Additionally, hiOL express paranodal proteins, such as neurofascin and ERMIN. However, the paranodal protein OPALIN is not expressed in hiOL suggesting that the interaction between axons and oligodendrocytes might be required for the upregulation of OPALIN. In their studies Jäckel et al. identified an oligodendroglial subcluster termed immune oligodendroglia (ImOLG) expressing among other markers CD74 and HLA-DRA which were enriched in MS brains [23]. hiOL from RRMS patients and controls express CD74 and HLA-DRA on protein level as well as numerous other immune-related proteins. However, we did not find any significant differences in the expression of these immune-associated proteins between hiOL from RRMS and controls suggesting that an involvement of oligodendrocytes in the immune response is not specific to RRMS hiOL.

Our data suggest that IFNγ, released by T cells, plays a major role in impairing oligodendroglial differentiation in MS but does not induce cell death. IFNγ and TNFα are highly expressed in MS lesions and earlier studies have shown that IFNγ and TNFα are able to induce oligodendroglial cell death [2, 51, 59]. This is in contrast to our data in which exposure to TNFα and IFNγ did not induce oligodendroglial cell death. These discrepancies might be due to species differences, differences in the cytokine concentrations, or distinct developmental stages of oligodendrocytes. Interestingly, recently published studies revealed that treatment with IFNγ enhanced expression of MHC class II proteins on murine OPCs as well as the presentation of antigens suggesting an active role of OPCs during inflammatory processes [15, 27]. Here, we show that an inflammatory environment mimicked by application of PBMC+ or recombinant IFNγ and TNFα also induces the expression of MHC class I and MHC class II proteins as well as several other immune markers on a transcriptional and translational level in hiOL further supporting the notion that oligodendrocytes may play a more active role in the immune response than it has been so far assumed. However, whether and how this contributes to the development and progression of MS remains to be determined.

Consistent with our finding that IFNγ significantly inhibits oligodendroglial differentiation, IFNγ has been reported to reduce developmental myelination and remyelination in the rodent CNS [33, 34]. Next to its detection in CNS tissue sections from patients with MS [65], IFNγ could be attributed to disease progression as its expression has been shown to be increased in the serum of MS patients with ongoing progression compared to patients without progression [25, 26]. Additionally, IFNγ has been connected to impaired differentiation of oligodendrocytes in rodents [1, 9]. Here, we provide the first evidence for an impaired oligodendroglial differentiation in humans caused by IFNγ. However, only the simultaneous application of recombinant IFNγ and TNFα impaired differentiation in hiOL, whereas IFNγ alone did not affect oligodendroglial differentiation. Blocking IFNγ alone, but not TNFα restored differentiation ability of hiOL supporting the major role of IFNγ for impaired oligodendroglial differentiation and suggesting that IFNγ itself is not sufficient but its presence is essential for immune cell-mediated oligodendroglial differentiation block. In line with this observation, the synergistic effects of IFNγ and TNFα have been reported [2, 7]. Interestingly, treatment with drugs that have been shown to promote oligodendroglial differentiation [12, 14, 41, 42], such as benztropine, miconazole, and clemastine was not able to rescue impaired oligodendroglial differentiation by PBMC+. We used drug concentrations that have been shown before to be efficient in promoting oligodendroglial differentiation; however, we cannot exclude that treatment with other concentrations may have resulted in a different outcome. Treatment of PBMCs prior to supernatant collection with the anti-inflammatory drug TF, which inhibits the proliferation of immune cells [4] and thus reduces the secretion of IFNγ, however, partly restores the differentiation of hiOL. The fact that TF treatment of PBMCs cannot fully restore hiOL differentiation might be explained with the residual IFNγ in PBMC+, as TF does not completely abolish IFNγ secretion in PBMCs. Nevertheless, our findings suggest that drug screenings, which take the inflammatory environment in MS lesions into account, might identify more potent remyelination-promoting compounds.

Our in vitro results demonstrate that IFNγ impairs oligodendroglial differentiation which might be one explanation among others for the negative outcome of a clinical pilot MS trial using IFNγ [45]. Our data could suggest the use of IFNγ inhibiting drugs to promote remyelination. However, blocking IFNγ enhanced disease severity and frequency of relapses in EAE mice [5, 21]. This might be explained by the fact that IFNγ displays dual roles in neuroinflammation. It is not only a proinflammatory cytokine causing damage in the CNS but also displays protective and regulatory roles [44]. Therefore, using neutralizing IFNγ as a treatment option in MS might only be successful in specific disease phases as could be shown in EAE [35]. Nevertheless, blocking of IFNγ induced pathways which inhibit oligodendroglial differentiation might support remyelination and clinical recovery in MS.

In summary, our data suggest that intrinsic oligodendroglial factors do not contribute to impaired remyelination in RRMS while extrinsic factors such as the inflammatory environment impair oligodendroglial differentiation and thereby might affect efficient remyelination. Thus, this data further supports the idea of MS as an immune-mediated rather than a degenerative disorder.

## Electronic supplementary material

Below is the link to the electronic supplementary material.

Supplementary file1 (PDF 2340 kb)

Supplementary file2 (XLSX 1148 kb)

Supplementary file3 (XLSX 1073 kb)
