# Supplementary material for: Extrinsic immune cell-derived, but not intrinsic oligodendroglial factors contribute to oligodendroglial differentiation block in multiple sclerosis
Source: Acta Neuropathol. 2020 Sep 7;140(5):715–36. doi: 10.1007/s00401-020-02217-8 (PMC7547031; doi:10.1007/s00401-020-02217-8)
Supplement: Supplementary file 1 — Supplementary file1 (PDF 2340 kb) [file 401_2020_2217_MOESM1_ESM.pdf]

Extrinsic immune cell-derived, but not intrinsic oligodendroglial factors contribute to oligodendroglial differentiation block in multiple sclerosis

Acta Neuropathologica

Laura Starost[1,2], Maren Lindner[3], Martin Herold[3], Yu Kang T. Xu[4], Hannes C.A. Drexler[5], Katharina Heß[1], Marc Ehrlich[1,2], Linda Ottoboni[6], Francesca Ruffini[6], Martin Stehling[2], Albrecht Röpke[7], Christian Thomas[1], Hans R. Schöler[2], Jack Antel[8], Jürgen Winkler[9], Gianvito Martino[6,10§], Luisa Klotz[3§], Tanja Kuhlmann[1*§]

[§] Shared last authorship

[1]Institute of Neuropathology, University Hospital Münster, 48149 Münster, Germany; [2]Department of Cell and Developmental Biology, Max Planck Institute for Molecular Biomedicine, 48149 Münster, Germany; [3]Department of Neurology with Institute of Translational Neurology, University Hospital Münster, 48149 Münster, Germany; [4]The Solomon H. Snyder Department of Neuroscience, Johns Hopkins University School of Medicine, Baltimore, MD 21205, USA; [5]Bioanalytical Mass Spectrometry, Max Planck Institute for Molecular Biomedicine, 48149 Münster, Germany; [6]Neuroimmunology Unit, Institute of Experimental Neurology, Division of Neuroscience, IRCCS San Raffaele Hospital, 20132 Milan, Italy; [7]Institute of Human Genetics, University of Münster, 48149 Münster, Germany; [8]Montreal Neurologic Institute, McGill University, Montreal, QC, Canada H3A 2B4; [9]Department of Molecular Neurology, University Hospital Erlangen, Friedrich-Alexander-Universität Erlangen-Nürnberg, 91054 Erlangen, Germany; [10]Vita Salute San Raffaele University, 20132 Milan, Italy.

*Corresponding author:
Tanja Kuhlmann, Institute of Neuropathology, University Hospital Münster, Pottkamp 2, 48149 Münster, Germany
Telephone +49 251 83 52614, Fax +49 251 83 56971
Email: tanja.kuhlmann@ukmuenster.de

SUPPLEMENTARY TABLES AND FIGURES

Table S1 MS patient brain sample cohort

	Number of tissue blocks	Number of patients (female to male)	Average age
Total	43	32 (23: 9)	48.75

Table S2 Overview of cell lines

Cell line	Group	Age at donation	Gender	Disease duration at timepoint of biopsy	Relationship	Treatment prior to biopsy	Relapse before biosy
C1	Control	32	Female	n.a.	monozygous twin	n.a.	n.a.
C2	Control	71	Male	n.a.	Non-relative	n.a.	n.a.
C3	Control	34	Female	n.a.	monozygous twin	n.a.	n.a.
RRMS1	RRMS	32	Female	11 years	monozygous twin	Rebif 44	30 days before biopsy
RRMS2	RRMS	36	Male	16 months	Non-relative	Rebif 22	16 months before biopsy
RRMS3	RRMS	34	Female	15 months	monozygous twin	Rebif 44	6 months before biopsy

Table S3 Antibodies applied in the study
ICC: immunocytochemistry, Neut: neutralization, FACS: flow cytometry, IHC: immunohistochemistry

Antibody	Dilution	Source	RRID	Application
Rabbit anti-NANOG	1:1000	Cell Signaling (4903)	AB_10559205	ICC
Mouse anti-TRA-1-60	1:100	Merck (MAB4360)	AB_2119183	ICC
Rabbit anti-OCT4	1:1000	Cell Signaling (2840)	AB_2167691	ICC
Mouse anti-SSEA4	1:100	Merck (FCMAB116P)	AB_528477	ICC

Goat anti-SOX1	1:150	R&D (AF3369)	AB_2239879	ICC
Mouse anti-NESTIN	1:300	R&D (MAB1259)	AB_2251304	ICC
Mouse anti-O4	1:500	R&D (MAB1326)	AB_357617	ICC
Rat-anti MBP	1:50	Abcam (AB7349)	AB_305869	ICC
Rabbit anti-Ki67	1:250	Abcam (AB16667)	AB_302459	ICC
Mouse anti-TUBBIII (TUJ1)	1:1000	Covance (MMS-435P)	AB_2313773	ICC
Mouse anti-SMA	1:100	Dako (M0851)	AB_2223500	ICC
Mouse anti-AFP	1:300	Dako (A0008)	AB_2650473	ICC
Ultra-LEAF™ Purified mouse-anti-IFN-γ	1:20	Biolegend (506532)	AB_2801092	Neut
LEAF™ Purified mouse-anti-TNF-α	1:20	Biolegend (502804)	AB_315252	Neut
Mouse-anti-O4-APC	1:50	Miltenyi (130-095-891)	AB_2751598	FACS
Mouse-anti-CD69-PE	1:200	Beckmann Coulter (IM1943U)	AB_2801272	FACS
Mouse-anti-CD8-PB	1:200	Biolegend (300928)	AB_10612929	FACS
Mouse-anti-CD74-PE	1:100	Biolegend (326808)	AB_2075505	FACS
Mouse-anti-CD54-PerCP/Cy5.5	1:100	Biolegend (353120)	AB_2715948	FACS
Mouse-anti-CD106-BV421	1:100	Biolegend (305816)	AB_2832596	FACS
Mouse-anti-CD14-BV510	1:200	Biolegend (301842)	AB_2561946	FACS
Mouse-anti-CD19-APC	1:200	Biolegend (302212)	AB_314242	FACS
Mouse-anti-CD4-BV510	1:200	Biolegend (317444)	AB_2561377	FACS
Mouse-anti-CD45-FITC	1:200	Biolegend (304006)	AB_314394	FACS
Mouse-anti-CD16-PB	1:200	Biolegend (302032)	AB_2104003	FACS
Mouse-anti-MHC-I (HLA-A,B,C)-AF700	1:100	Biolegend (311438)	AB_2566306	FACS

Mouse-anti-MHC-II (HLA-DR)-PE	1:200	BD Biosciences (555812)	AB_396146	FACS
Mouse-anti-MHC-II (HLA-DR)-APC	1:200	BD Biosciences (559868)	AB_399974	FACS
Mouse anti- MHC-II (HLA-DR)-FITC	1:100	Biolegend (307604)	AB_314682	FACS
Mouse-anti-CD20	1:700	Dako (M0755)	AB_2282030	IHC
Mouse-anti-CD68	1:200	Dako (M0814)	AB_2314148	IHC
Mouse-anti-CD3	1:25	Dako (M7254)	AB_2631163	IHC
Rabbit-anti-MBP	1:1000	Dako (A0623)	AB_2650566	IHC
Mouse-anti-NF	1:1000	Dako (M0762)	AB_2314899	IHC

Table S4 List of applied primers

Primer	Sequence
OCT4 endo	fwd: 5'- GGA AGG AAT TGG GAA CAC AAA GG -3'<br>rev: 5' - AAC TTC ACC TTC CCT CCA ACC A -3'
SOX2 endo	fwd: 5'- TGG CGA ACC ATC TCT GTG GT -3'<br>rev: 5'- CCA ACG GTG TCA ACC TGC AT -3'
NANOG	fwd: 5'- CCT GTG ATT TGT GGG CCT G -3'<br>rev: 5'- GAC AGT CTC CGT GTG AGG CAT -3'
B2M	fwd: 5’- GAT GAG TAT GCC TGC CGT GT -3’<br>rev: 5’- CTG CTT ACA TGT CTC GAT CCC A -3’
TAP1	fwd: 5’- TTT GAG TAC CTG GAC CGC AC -3’<br>rev: 5’- AAT GTC AGC CCC TGT AGC AC -3’
CD74	fwd: 5’- TGA TGC ACC TGC TCC AGA -3’<br>rev: 5’- CCC AGA TCC TGC TTG GTC -3’
ICAM-1	fwd: 5’- TCT TCC TCG GCC TTC CCA TA -3’<br>rev: 5’- AGG TAC CAT GGC CCC AAA TG -3’
VCAM-1	fwd: 5’- GGG AAG CCG ATC ACA GTC AA -3’<br>rev: 5’- GGG ACT TCC TGT CTG CAT CC -3’
GAPDH	fwd: 5’- CTG GTA AAG TGG ATA TTG TTG CCA T -3’<br>rev: 5’- TGG AAT CAT ATT GGA ACA TGT AAA CC -3’

Table S5 Proteins expressed in hiOL associated with GO terms “immune response” (see separate file)

Table S6 Proteins expressed in iPSC-derived oligodendrocytes generated by an *in vitro* patterning approach associated with GO terms “immune response” (see separate file)

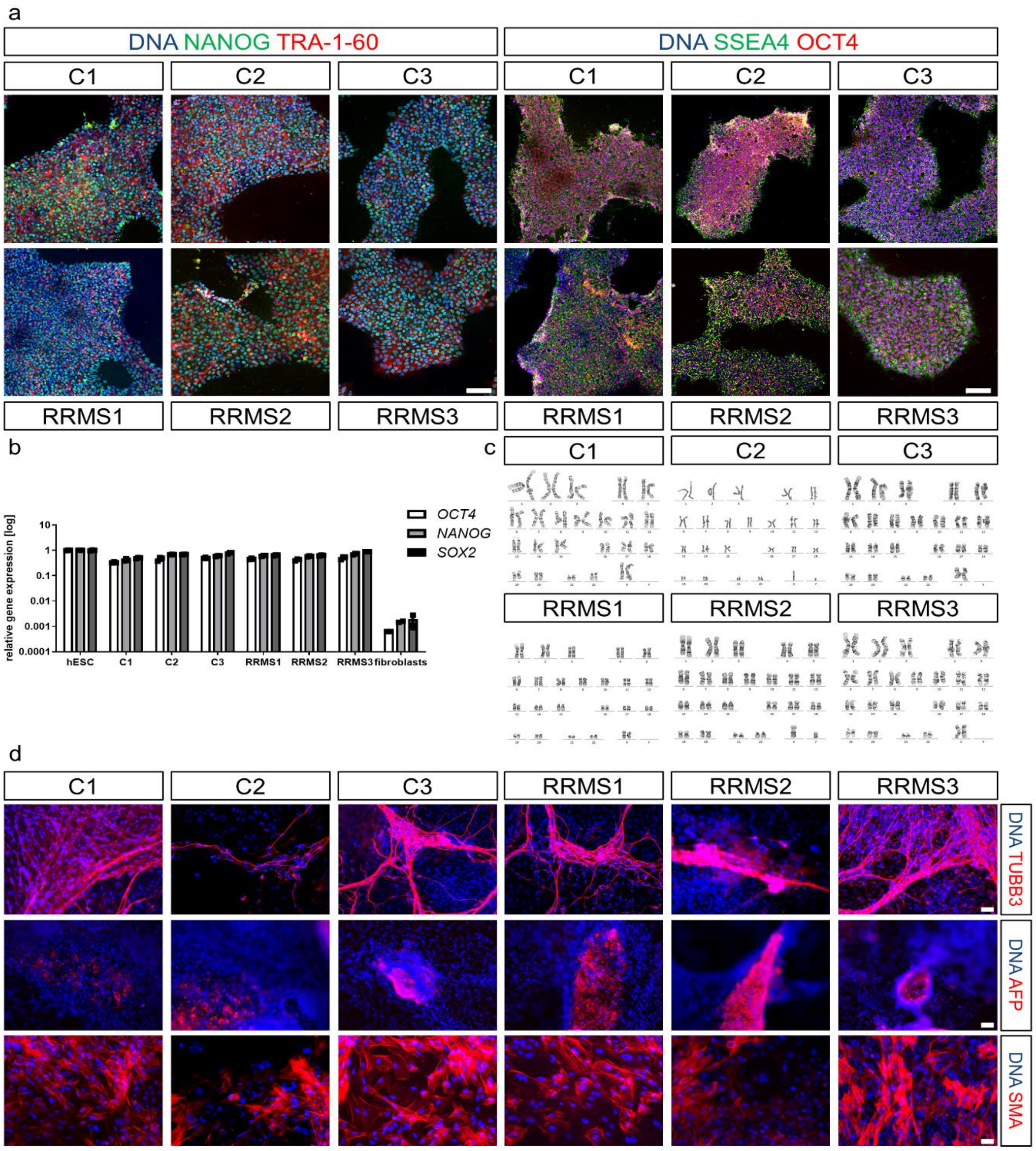

Fig. S1 RRMS and control fibroblasts were successfully reprogrammed into iPSCs

a Representative ICC of RRMS and control iPSCs stained for pluripotency markers NANOG (green) and TRA-1-60 (red) or SSEA-4 (green) and OCT4 (red) indicating expression of pluripotency markers on protein level. **b** Expression of pluripotency genes *OCT4, NANOG,* and *SOX2* in RRMS and control iPSCs. Expression levels are similar to those in human embryonic stem cells (hESCs) and increased compared to fibroblasts indicating successful reprogramming of iPSCs. **c** Karyogram analysis of RRMS and control iPSCs demonstrating normal karyotypes for all lines. **d** Representative ICC of RRMS and control iPSCs after *in vitro* differentiation into cells of all three germ layers. RRMS and control iPSC express marker for ectoderm (TUBB3, red), endoderm (AFP, red), and mesoderm (SMA, red).

Data (**b**) are presented as mean of three technical replicates +SEM.

Scale bars: 50 µm. DAPI was used to counterstain the nuclei.

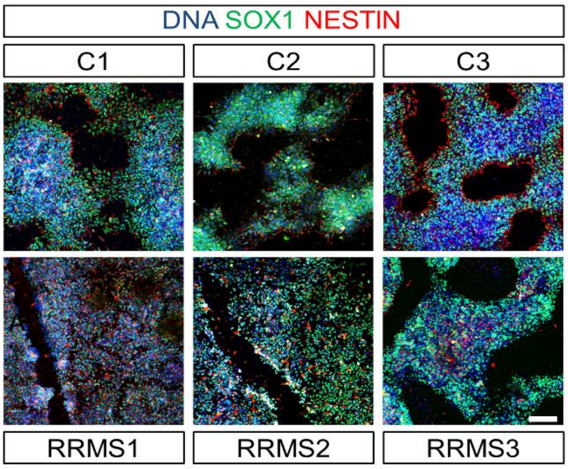

Fig. S2 RRMS and control NPCs were successfully generated

Representative ICC of RRMS and control NPCs stained for NPC markers SOX1 (green) and NESTIN (red).

Scale bar: 50 µm. DAPI was used to counterstain the nuclei.

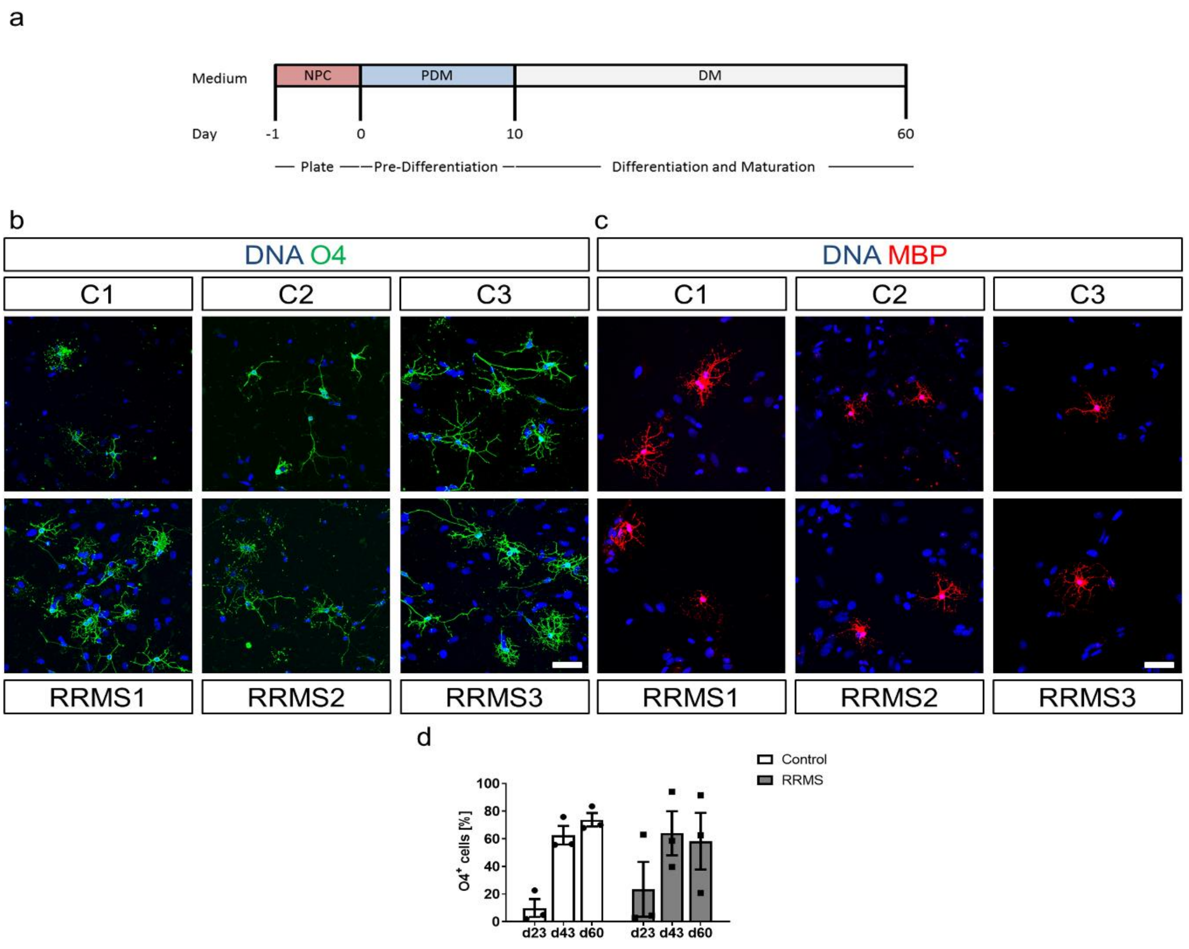

Fig. S3 RRMS and control NPCs show the same ability to differentiate into iPSC-derived oligodendrocytes by using an *in vitro* patterning approach

a Schematic presentation of the applied *in vitro* patterning protocol. NPC = NPC medium; PDM = Pre-Differentiation medium; DM = Differentiation medium **b, c** Representative ICC for O4 (green, **b**) and MBP (red, **c**) at day 43 (**b**) or 60 (**c**) of differentiation demonstrating the differentiation of RRMS and control NPCs into oligodendrocytes using an *in vitro* patterning protocol. **d** Flow cytometry-based quantification of $O4^+$ cells at day 23, 43, and 60 of differentiation indicating no significant differences between RRMS and control iPSC-derived oligodendrocytes (n=3).

Data are presented as mean +SEM. Statistical significance was determined by Bonferroni-corrected two-way ANOVA.

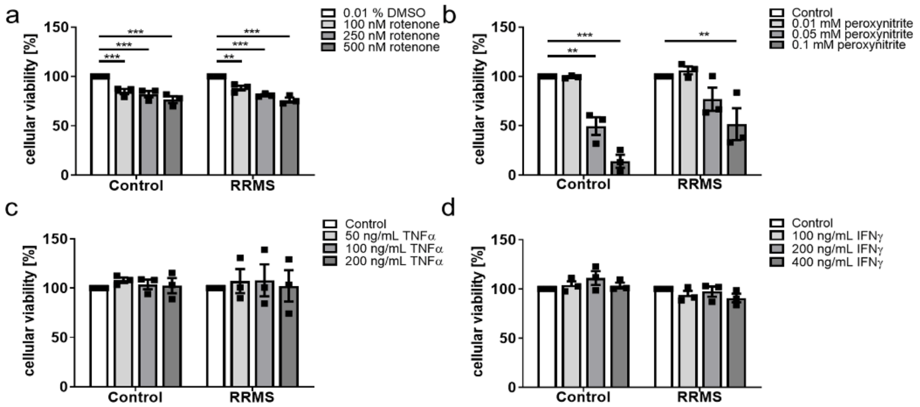

Fig. S4 RRMS and control hiOL display a similar stress response

a-d Quantification of cellular survival of RRMS and control hiOL after application of different stressors by luminescent measurement of intracellular ATP levels by CellTiterGlo. Different concentrations of rotenone (100, 250, 500 nM) (**a**), peroxynitrite (0.01, 0.05, 0.1 mM) (**b**), TNFα (50, 100, 200 ng/mL) (**c**), and IFNγ (100, 200, 400 ng/mL) (**d**) were applied for 24 (**a**, **b**) or 48 h (**c**, **d**) to $O4^+$ RRMS and control hiOL sorted at day 21 of differentiation. Oxidative stress induced by rotenone (**a**) and peroxynitrite (**b**) results in significantly impaired survival but no significant differences between RRMS and control hiOL. Inflammatory cytokines TNFα (**c**) and IFNγ (**d**) do not show any impact on cellular survival (n=3).

Data are presented as mean +SEM. Statistical significance was determined by Bonferroni-corrected two-way ANOVA (**p < 0.01, ***p < 0.001).

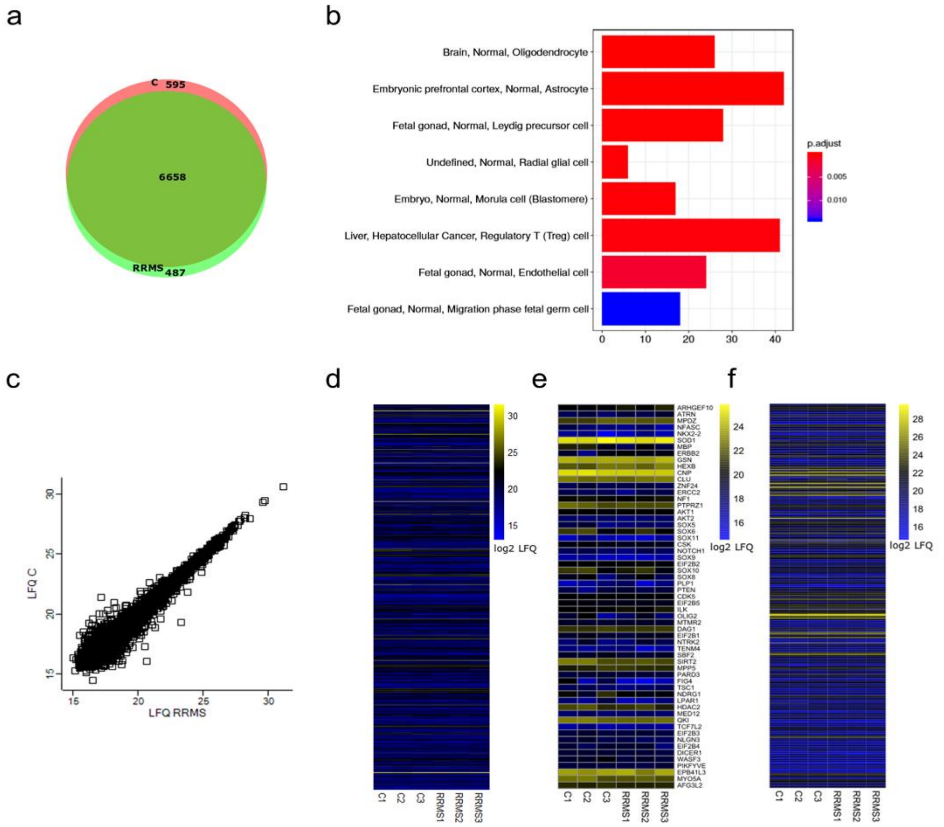

Fig. S5 iPSC-derived oligodendrocytes from RRMS patients and controls generated by an *in vitro* patterning approach display highly similar proteomes

a Venn-Diagram showing proteins detected only in pooled control iPSC-derived oligodendrocytes (C), pooled RRMS iPSC-derived oligodendrocytes (RRMS) or both indicating that the majority of proteins is shared between both groups. **b** Cell marker enrichment analysis of proteins whose LFQ intensities account for the top 10 % of all LFQ intensities across the whole data set demonstrating that proteins characteristic for oligodendrocytes are significantly enriched in the data set. **c** Scatter plot of log2 transformed LFQ intensities of control and RRMS iPSC-derived oligodendrocytes indicating a strong correlation between the proteomes of RRMS and control iPSC-derived oligodendrocytes. **d** HeatMap of all identified proteins based on log2 transformed LFQ intensities indicating that protein expression is highly similar between RRMS and control iPSC-derived oligodendrocytes. **e** HeatMap of log2 transformed LFQ intensities for proteins associated with GO terms “central nervous system myelination”, “myelin maintenance”, “myelin assembly”, “myelination”, “oligodendrocyte cell fate specification”, “oligodendrocyte development”, “oligodendrocyte differentiation”, and “positive regulation of oligodendrocyte differentiation” demonstrating presence and similar expression of oligodendroglial proteins in all analyzed samples. **f** HeatMap of log2 transformed LFQ intensities for proteins associated with GO term “immune response” indicating presence and similar expression levels of proteins connected to the immune system in all analyzed samples.

Proteomic analysis was performed in two independent experiments with three RRMS patients and three healthy controls including in total two different NPC clones per patient and healthy control. Statistical significance was determined by Student’s *t*-test with permutation based FDR.

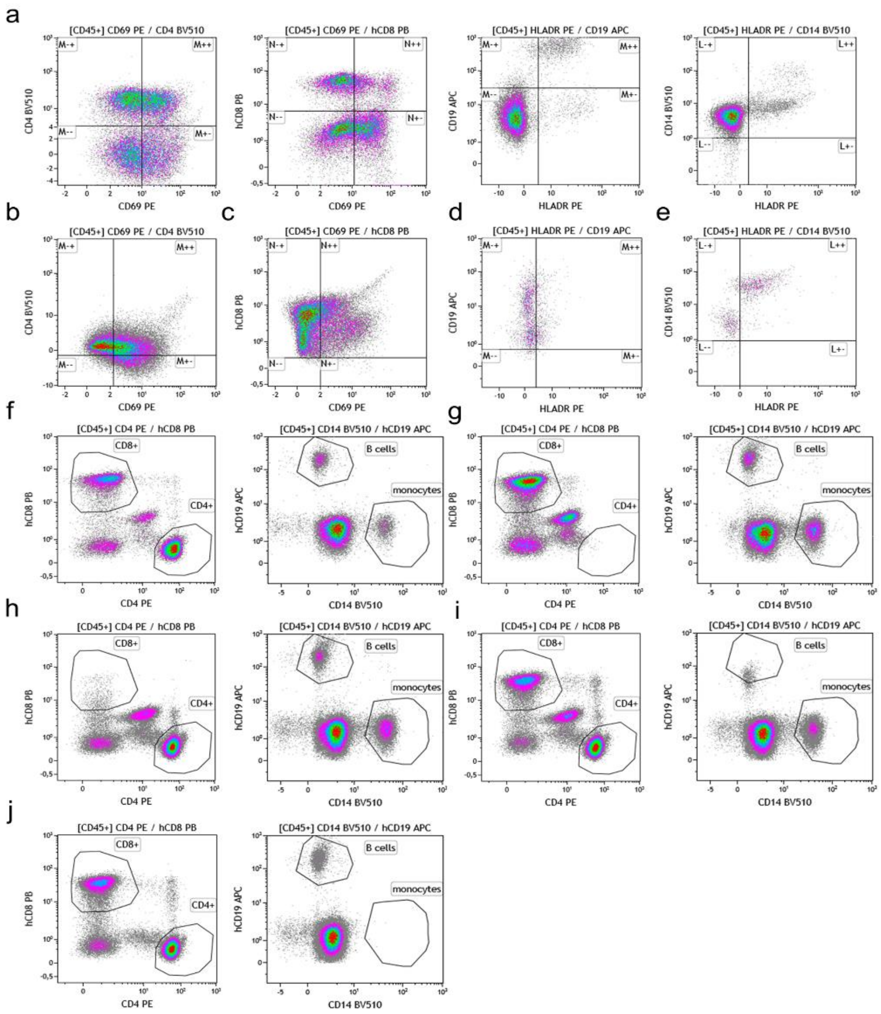

Fig. S6 Activation of PBMCs, T cells, monocytes, and B cells after treatment with PHA and depletion of distinct immune cell populations from PBMCs

a-e Representative flow cytometry-plots of $CD4^{+}$ T cells, $CD8^{+}$ T cells, $CD19^{+}$ B cells, and $CD14^{+}$ monocytes stimulated with 5 µg/mL PHA either in whole PBMCs (**a**) or after isolating single immune cell populations (**b-e**). Plots indicate presence of activation markers CD69 or HLA-DR after treatment with PHA for 48 h. **f-j** Representative flow cytometry-plots for immune cell type markers CD4, CD8, CD14 and CD19 in PBMCs (**f**) and after depletion of $CD4^{+}$ T cells (**g**), $CD8^{+}$ T cells (**h**), $CD19^{+}$ B cells (**i**), or $CD14^{+}$ monocytes (**j**) showing successful depletion of respective immune cell populations.

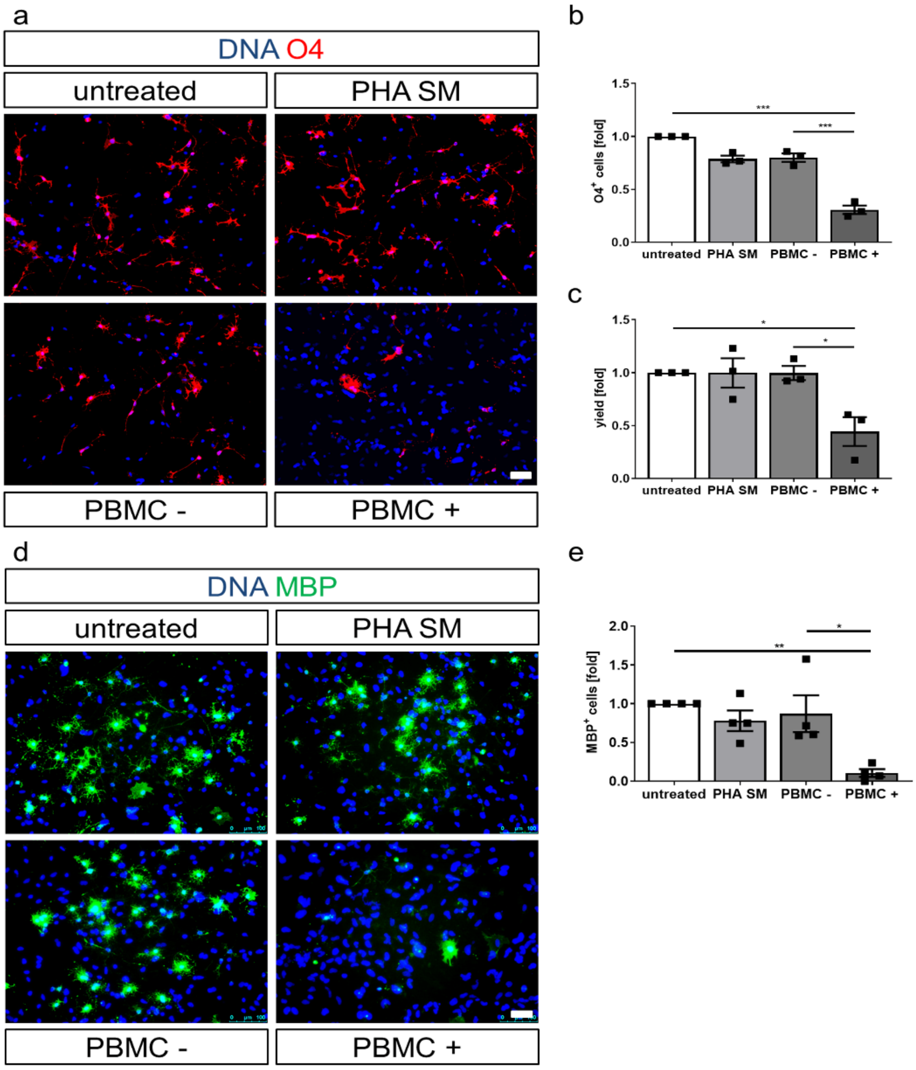

Fig. S7 Supernatants of activated PBMCs significantly inhibit the differentiation of RRMS hiOL
a Representative ICC of $O4^{+}$ (red) hiOL of RRMS patients that were either left untreated or treated with PHA SM or PBMC –/+ from day 4 until day 21. **b, c** Flow cytometry-based quantification of $O4^{+}$ hiOL at day 21 of differentiation indicating a significantly decreased percentage (**b**) and yield (**c**) of $O4^{+}$ hiOL by PBMC + (n=3). **d** Representative ICC of MBP^{+} (green) hiOL at day 35 of differentiation. Untreated cells were sorted by flow cytometry for O4 at day 21 of differentiation and subsequently either left untreated or treated with PHA SM, PBMC -, or PBMC + until day 35. **e** Quantification of MBP^{+} hiOL at day 35 of differentiation by ICC after sorting of untreated $O4^{+}$ hiOL by flow cytometry at day 21 displaying a significantly impaired differentiation into MBP after treatment with PBMC + (n=4).
Data are presented as mean +SEM. Statistical significance was determined by Bonferroni-corrected one-way ANOVA (*$p < 0.05$, **$p < 0.01$, ***$p < 0.001$). The relative number of $O4/MBP^{+}$ cells present under untreated conditions (**b**, **c**, **e**) was arbitrarily set to 1 and used to normalize in pairwise fashion. Scale bars: 50 µm; DAPI was used to counterstain the nuclei.

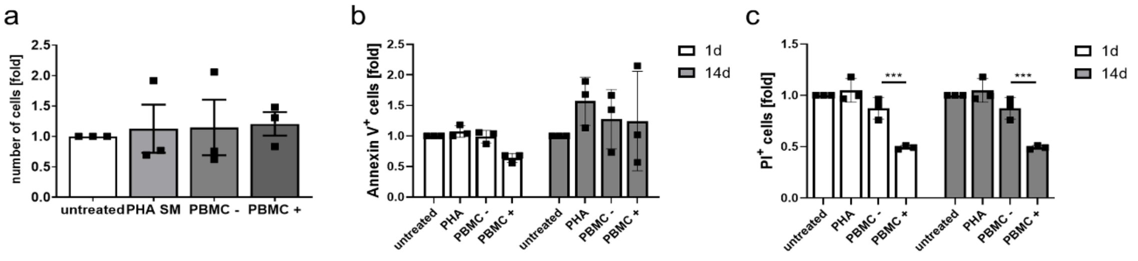

Fig. S8 Application of supernatants of activated PBMCs does not induce cell death in hiOL

a Total amount of cells detected by flow cytometry at day 21 of differentiation into hiOL indicating that cell numbers were not affected by treatment with PHA SM, PBMC -, or PBMC + (n=3). **b** Flow cytometry-based quantification of the percentage of Annexin V^+ cells one day or 14 days after application of PHA SM, PBMC -, or PBMC + demonstrating no significant effect on the percentage of apoptotic cells after application of PBMC + (n=3). **c** Flow cytometry-based quantification one day or 14 days after application of PHA SM, PBMC -, or PBMC + revealing significantly decreased percentages of PI^+ dying cells by PBMC + (n=3),

Data are presented as mean +SEM (***p < 0.001). Statistical significance was determined by Bonferroni-corrected one-way ANOVA. The relative number of Annexin V/PI^+ cells present under untreated conditions was arbitrarily set to 1 and used to normalize in pairwise fashion.

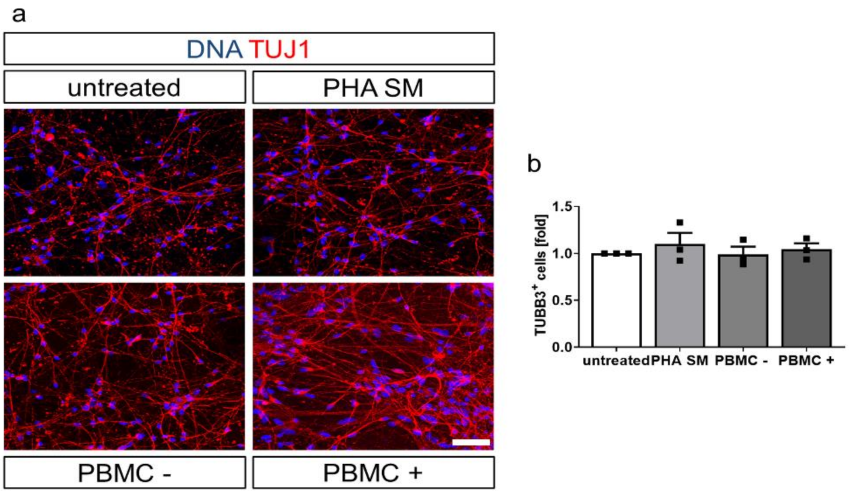

Fig. S9 Application of supernatants of activated PBMCs does not impair the differentiation into iPSC-derived neurons

a Representative ICC of TUJ1^{+} (red) iPSC-derived neurons of healthy donors that were either left untreated or treated with PHA SM, PBMC –, or PBMC +. Cultures were treated from day 6 until day 23 with supernatants and staining was performed on day 23. DAPI was used to counterstain the nuclei. Scale bar: 50 µm **b** Quantification of TUJ1^{+} iPSC-derived neurons at day 23 of differentiation by ICC indicating that differentiation into neurons is not affected by application of PBMC + (n=3).

Data are presented as mean +SEM. Statistical significance was determined by Bonferroni-corrected one-way ANOVA. The relative number of TUJ1^{+} cells present under untreated conditions was arbitrarily set to 1 and used to normalize in pairwise fashion.

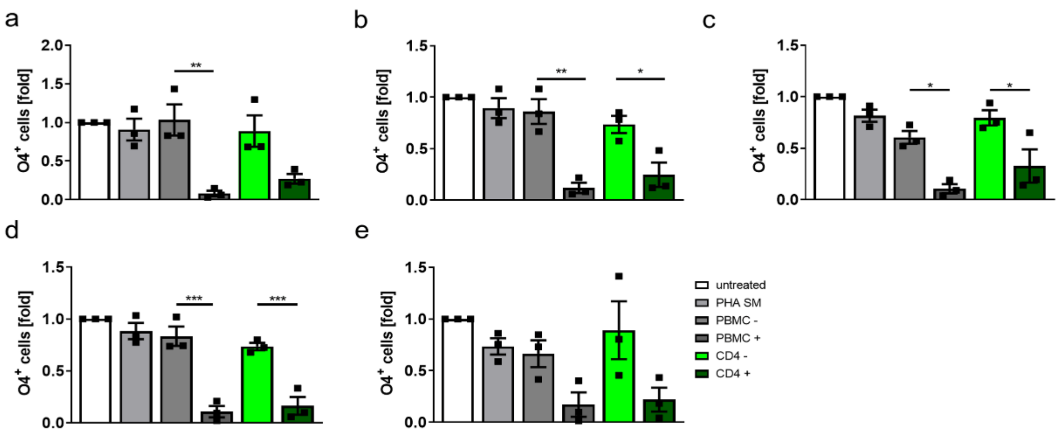

Fig. S10 Supernatants of activated CD4^{+} T cells from different donors impair the differentiation into hiOL

a-e Flow cytometry-based quantification of O4^{+} hiOL at day 21 of differentiation. Cells were either left untreated or treated with PHA SM, PBMC -/+, or CD4 -/+ showing that PBMC + and CD4 + from different donors lead to a significantly impaired differentiation into O4^{+} cells (n=3).

Data are presented as mean +SEM. Statistical significance was determined by Bonferroni-corrected one-way ANOVA (*p < 0.05, **p < 0.01, ***p < 0.001). The relative number of O4^{+} cells present under untreated conditions was arbitrarily set to 1 and used to normalize in pairwise fashion.

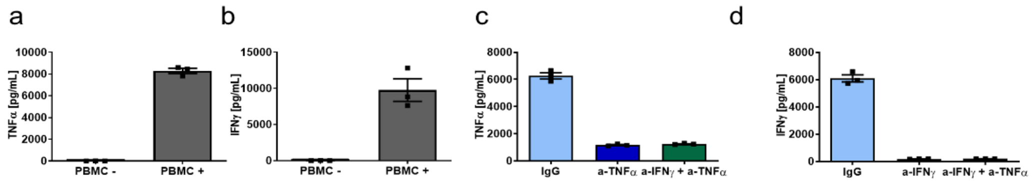

Fig. S11 Supernatants of activated PBMCs contain high concentrations of IFNγ and TNFα

a-d Concentrations of TNFα (**a, c**) and IFNγ (**b, d**) in the supernatants of PBMC -/+ (**a**, **b**) or in PBMC + that were either treated with IgG control, a-TNFα, a-IFNγ, or combination of both (**c**, **d**) as determined by ELISA indicating an increased concentration of TNFα and IFNγ after activation of PBMCs and decreased concentrations after using neutralizing antibodies.

Data are presented as mean of three technical replicates +SEM.

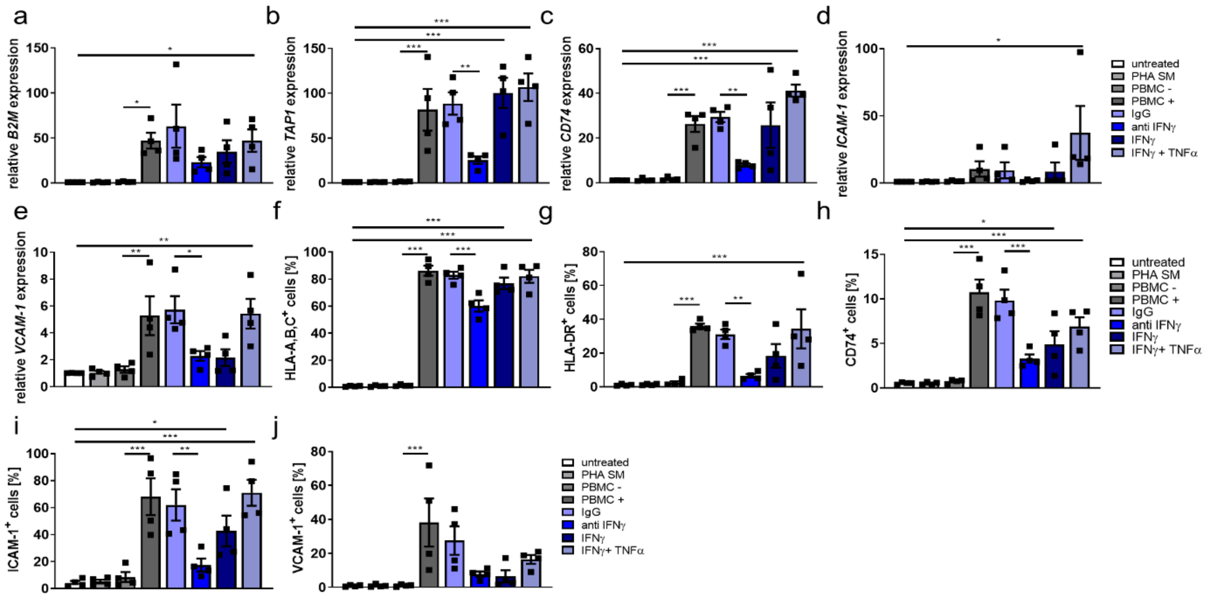

Fig. S12 Supernatants of activated PBMCs induce the expression of immune markers in hiOL via IFNγ secretion

a-e Expression of immune markers *B2M* (**a**), *TAP1* (**b**), *CD74* (**c**), *ICAM-1* (**d**), and *VCAM-1* (**e**) on transcriptional level after application of PHA SM, PBMC -/+, PBMC + which were either incubated with IgG or a-IFNγ beforehand, recombinant IFNγ, or combination of recombinant IFNγ and TNFα to differentiating hiOL from day 4 until day 21 of differentiation (n=4). Application of PBMC + and the combination of recombinant IFNγ and TNFα result in significantly enhanced transcript levels of immune markers. Enhanced transcripts are decreased when PBMC + were incubated with a-IFNγ before compared to IgG control. **f-j** Flow cytometry-based quantification of immune markers HLA-A,B,C (**f**), HLA-DR (**g**), CD74 (**h**), ICAM-1 (**i**), and VCAM-1 (**j**) on protein level after application of PHA SM, PBMC -/+, PBMC + which were either incubated with IgG or a-IFNγ beforehand, recombinant IFNγ, or combination of recombinant IFNγ and TNFα to differentiating hiOL from day 4 until day 21 of differentiation (n=4). Application of PBMC + and the combination of recombinant IFNγ and TNFα significantly increase protein levels of immune markers. Enhanced presence of markers is decreased when PBMC + were incubated with a-IFNγ before compared to IgG control.

Data are presented as mean +SEM. Statistical significance was determined by Bonferroni-corrected one-way ANOVA (*$p < 0.05$, **$p < 0.01$, ***$p < 0.001$).

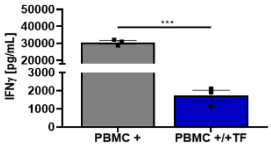

Fig. S13 Incubation of PBMCs with TF during activation results in significantly reduced IFNγ secretion

Concentration of IFNγ in the supernatants of PBMC + with and without treatment with TF during activation determined by ELISA indicating significantly decreased concentration of IFNγ after treatment with TF (PBMC +/+TF) compared to untreated PBMC + (PBMC +).

Data are presented as mean +SEM. Statistical significance was determined by two-tailed Student's *t*-test (***$p < 0.001$).
